# A review of *Leishmania* infections in American Phlebotomine sand flies – Are those that transmit leishmaniasis anthropophilic or anthropportunists?★

**DOI:** 10.1051/parasite/2025039

**Published:** 2025-09-08

**Authors:** Jeffrey Jon Shaw

**Affiliations:** Parasitology Department, São Paulo University 1374 Av. Prof. Lineu Prestes São Paulo State of São Paulo 05508-000 Brazil

**Keywords:** *Leishmania*, Infections, American sand flies, Feeding habits

## Abstract

Understanding why Diptera, such as mosquitoes and sand flies, feed on humans is crucial in defining them as vectors of diseases such as malaria, dengue fever, Zika virus, and leishmaniasis. Determining their attraction to humans (anthropophily) helps in assessing the risk of disease transmission, designing effective vector control strategies, and monitoring the effectiveness of existing control measures. An important question is whether they are specifically attracted to humans in preference to other mammals or whether there is something else at play. In this paper, I propose that the idea that saying species are “anthropophilic” when they are preferentially attracted to humans is misleading and that such species are generalists that will opportunistically feed on a wide range of animals including humans when they are available. Other species are specialists that, under rare circumstances, accidently feed on humans. For these groups, I propose the names anthropportunists and anthroaccidentalists, respectively. The level of contact depends on a range of environmental factors and it is these that must be considered in evaluating potential vector importance and management. In this paper, I propose a review of the *Leishmania* identified in American sand flies and relate them to these two concepts and how it is linked to taxonomic groups, evolution, and ecology. There are records of *Leishmania* in 91 species, which is only 16.5% of American sand fly species. Of these infections, 56.7% are in the genera *Lutzomyia, Nyssomyia, Pintomyia*, and *Psychodopygus*, which are typically generalist anthropportunists. Of the species considered to be proven vectors, 77.4% belong to these four genera. As infections were detected by a variety of methods, further case-by-case field studies are required to evaluate the vectorial role of many of the sand fly species in which *Leishmania* have been found.

## Abstract (fr)

Comprendre pourquoi les diptères, tels que les moustiques et les phlébotomes, se nourrissent de l’homme est essentiel pour les définir comme vecteurs de maladies telles que le paludisme, la dengue, le virus Zika et la leishmaniose. Déterminer leur attirance pour l’homme (anthropophilie) permet d’évaluer le risque de transmission de maladies, de concevoir des stratégies efficaces de lutte antivectorielle et de surveiller l’efficacité des mesures de contrôle existantes. La question est de savoir s’ils sont spécifiquement attirés par l’homme plutôt que par d’autres mammifères, ou existe-t-il un autre concept sous-jacent ? Dans cet article, je propose que l’idée selon laquelle les espèces sont préférentiellement attirées par l’homme lorsqu’on les qualifie d’« anthropophiles » est trompeuse et que ces espèces sont généralistes et se nourrissent opportunément d’un large éventail d’animaux, y compris l’homme lorsqu’il est disponible. D’autres sont des spécialistes qui, dans de rares cas, se nourrissent accidentellement de l’homme. Pour ces groupes, je propose respectivement les termes anthropoportunistes et anthropoaccidentels. Le niveau de contact dépend de divers facteurs environnementaux, et ce sont ces derniers qui doivent être pris en compte pour évaluer l’importance potentielle du vecteur et sa gestion. Cet article passe en revue les *Leishmania* identifiées chez les phlébotomes américains et les relie à ces deux concepts, ainsi qu’à leurs liens avec les groupes taxonomiques, l’évolution et l’écologie. On a recensé des *Leishmania* dans 91 espèces, soit seulement 16,5 % des espèces de phlébotomes américains. Parmi ces infections, 56,7 % concernent les genres *Lutzomyia*, *Nyssomyia*, *Pintomyia* et *Psychodopygus*, qui sont typiquement anthropoportunistes généralistes. Parmi les espèces considérées comme vecteurs avérés, 77,4 % appartiennent à ces quatre genres. Les infections ayant été détectées par diverses méthodes, des études de terrain supplémentaires, au cas par cas, sont nécessaires pour évaluer le rôle vectoriel de nombreuses espèces de phlébotomes chez lesquelles des *Leishmania* ont été trouvées.

## Introduction

Anthropophily in dipteran biology signifies an attraction or preference for humans, feeding on humans, breeding in anthropogenic environments, and a habitat preference for these same biomes. Because of the feral origin of blood-sucking Diptera, it is unwise to consider that they are preferentially attracted to humans, as their main food sources are non-human vertebrates. However, attraction to anthropogenic biomes can enhance breeding and this closeness to humans, in the absence of other mammals, means that humans become the principal food source, confirmed by blood meal identifications.

In the literature, there are phrases such as “At least 15 *Lutzomyia* spp. are known to be anthropophilic and are strongly suspected as vectors”, but the criteria for such assertions are not mentioned nor what species they are. In others, there are statements such as “biting rates”, but they refer to catches in traps. These records may refer to species found to be biting humans, taken when landing on a protected individual or captured in Shannon traps. Sometimes, Shannon trap catches are equated to anthropophily. However, this may be problematic because there is sometimes a light source in this type of trap. Photophilia varies between species. In one study [56], the proportion of *Nyssomyia yuilli* and *Lutzomyia tortura* captured landing on humans was 1/3.3 and taken in a Shannon trap was 1/0.005. This indicates that Shannon trap catches reflected *Ny. yuilli*’s attraction for humans, but not *Lu. tortura*’s, and results with CDC traps showed that *Ny. yuilli* is attracted to light but *Lu. tortura* is not. This study emphasized that one trapping method may be useful for evaluating attraction of one species to humans, but for others, it is not. Also, it cannot be assumed that being attracted to humans is synonymous with “feeding on humans”, sometimes referred to a human-biting. I only use the term human-biting when it refers to observations of flies physically biting humans. Given the antiquity of sand flies and their co-evolution with mammalian orders, it is difficult to accept the evolution of specific attraction to humans. As a result, we need to reset the idea of sand fly “anthropophily” in a more realistic manner that relates to these insects’ feeding preferences, which have guaranteed their survival, and considering that the species evolved together with vertebrate groups, including mammals. Based on the records of Leishmaniinae infections, this research questions the usefulness of “anthropophily” to describe the feeding habits of American sand flies, as well as confusing derivatives, such as “anthropo-zoophilic” and “zoo-anthropophilic” that appear in the literature. Other researchers have replaced anthropophilic with terms such as human-feeding tendency, opportunistic feeder, or eclectic feeder. These are improvements but they do not dissect the biological complexity that is associated with different feeding habits.

In relation to the above, I am introducing the use of the “Generalist” and “Specialist” concepts to describe host preferences, how they differ, and their epidemiological implications:

**Generalists and Specialists**:

**1. Host range:**

***Generalist sand flies***: feed on a wide range of hosts and are adaptable and readily switch between available hosts for blood meals.

***Specialist sand flies****:* have more restricted host ranges and are highly adapted to specific hosts or host groups and environments.

**2. Adaptations:**

***Generalist sand flies***: have adaptations that allow them to exploit a variety of hosts. They may have specialized mouthparts that can pierce the skin of different types of animals, as well as mechanisms to detect hosts and avoid host defences.

***Specialist sand flies***: are often highly adapted to feed on specific hosts. Their mouthparts may be specialized to penetrate the skin of particular animals, and they may possess sensory organs that help them locate their preferred hosts.

**3. Transmission:**

***Generalist sand flies***: may support the multiplication and transmission of different parasites that results in the spread of diseases to various host species. Some have been considered “permissive” vectors. This has occurred with visceral leishmaniasis in the Americas.

***Specialist sand flies***: transmission is often more focused on specific hosts or host groups, potentially playing key roles in transmitting pathogens and parasites within certain ecosystems, such as diseases affecting livestock or wildlife.

**4. Ecological role:**

***Generalist sand flies***: can have a significant impact on the health of both human and animal populations by being able to breed in anthropogenic environments. Their presence can influence disease transmission dynamics and affect the behavior and potentially the distribution of their hosts.

***Specialist sand flies***: may have a more localized impact on specific host populations or ecosystems. They may be important vectors of diseases within their specialized host groups and can influence the health and behavior of those hosts.

**5. Evolution:**

***Generalist sand flies***: their evolution is linked to environments where there are multiple blood sources.

***Specialist sand flies***: their evolution is linked to more specific environments that favor close relationships with specific hosts.

Additionally, I propose three subdivisions within the above categories of generalists and specialists that are orientated towards their behavior in seeking humans as a food source. These subdivisions are anthropportunistic, anthroaccidentalistic, and zoophilic. The first two are new terms and the other is widely used in biology. Evaluating these categories is complex and is based on many parameters that range from how flies were collected to molecular identification of blood meals. Species considered to belong to any one of these categories can be generalists or specialists. Some generalists, when feeding, transmit Leishmaniinae parasites to a wide of hosts belonging to different mammalian orders and on occasions opportunistically feed on humans. Feeding on what is available is an excellent survival strategy and for those that are known to bite humans, I use the term anthropportunists. They are attracted to humans, may or may not be the dominant species in light and Shannon traps, and are associated with the dominant *Leishmania* species found in humans. There are others with more focused ecological roles within ecosystems and only under unusual circumstances bite people. I refer to these as anthroaccidentalists. They contribute minimally to zoonotic transmission of *Leishmania* species that are less common in humans. The categorization of species as zoophilic is based on absence of any information of their contact with people. They may be either generalists or specialists and may or may not play a role in the enzootic cycle. Generalists and specialists may belong to any one of these three categories. Assignment to these different categories was based on host preferences, trapping methods, feeding behavior, leishmanial infections, ecological adaptability, and epidemiological importance. The use of anthropophily and anthrophilic is limited to being found in anthropogenic biomes.

In 1940, Pessôa and Pestana [93] recorded the first flagellate infection in *Migonemyia migonei.* The earlier records between 1940 and 1965, as well as similar more recent ones, are not cited in this paper because there were no specific parasite identifications. However, infections in species mentioned in these publications have subsequently been identified, confirming identifications based on circumstantial epidemiological evidence. A review of the literature between 1965 and 2024 identified *Leishmania* infections in 91 sand fly species. These are listed in Table 1 together with their different behavioral character categories mentioned above, and the method of detection. These infections were reported over a period of 59 years using a variety of methods ranging from identifying parasites isolated from dissected flies to indirect molecular detection. However, they do not relate to vectorial capacity nor to the number of times that a species has been found to be infected. The literature citation refers to the first time the infections were published. At the time of writing, there are approximately 550 named phlebotomine species (Galati personal communication) in the Americas. This means that so far *Leishmania* have only been found in 16.4% of the American sand fly species. Of the 91 mentioned above, 75 were considered to be generalists or 16 as specialists and 51 as anthropportunists, 15 as anthroaccidentalists, 24 as zoophilic and one as phytophagous. This potentially suggests that if such infections were transmissible, 42.2% of the species would not represent a serious threat of zoonotic transmission. Older works refer to *in vivo* dissections coupled with biochemical identifications and even experimental transmissions. As a result, they more accurately indicate transmissibility and vectorial status, but in the absence of such observations, many recent molecular identifications merely indicate possible roles and nothing more. A method identifying metacyclics would add weight to the potential importance of infections detected by molecular techniques. The data also showed that 56.7% of infected species belonged to the genera *Lutzomyia, Nyssomyia, Pintomyia*, and *Psychodopygus.* This emphasizes the epidemiological importance of species belonging to these genera, especially as some species of the first three-genera breed in anthropogenic biomes.

**Table 1:** The feeding habits and *Leishmania* infections of American Phlebotomine species.

	Ha	Fe	Genus	Species	*Leishmania* parasite
1	S	Ac	*Bichromomyia*	***flaviscutellata***	***L.* (*L*.) *ama*** [62]^**D**^; *L.* (*V*.) *bra* [140]^**M**^
2	S	Ac	*Bichromomyia*	***olmeca***	*L.* (*L*.) *ama* [45]^**D**^; ***L.* (*L*.) *mex*** [11]^**D**^
3	S	Ac	*Bichromomyia*	*reducta*	*L.* (*L*.) *ama* [45]^**D**^
4	S	Ph	*Brumptomyia*	*leopoldoi*	*L.* (*M*.) sp. [98]^**M**^
5	S	Zo	*Dampfomyia*	***anthrophora***	***L.* (*L*.) *mex*** [78]^**M**^
6	G	Zo	*Evandromyia*	*apurinan*	*L.* (*V*.) *bra* [127]^**M**^
7	G	Zo	*Evandromyia*	*cortelezzii*	*L.* (*L*.) *inf* [19]^**M**^; *L.* (*V*.) *bra* [112]^**M**^
8	G	Zo	*Evandromyia*	*edwardsi*	*L.* (*V*.) *bra* [131]^**D**^
9	G	Zo	*Evandromyia*	*infraspinosa*	*L.* (*V*.) *bra* [24]^**M**^
10	G	Zo	*Evandromyia*	*lenti*	*L.* (*L*.) *inf* [105]^**M**^; *L.* (*V*.) *bra* [87]^**M**^
11	G	Zo	*Evandromyia*	*salesi*	*L.* (*L*.) *inf* [121]^**D**^; *L.* (*V*.) sp. [20]^**M**^
12	G	Zo	*Evandromyia*	*saulensis*	*L.* (*V*.) *bra* [3]^**M**^
13	G	Zo	*Evandromyia*	*termitophila*	*L.* (*L*.) *inf* [120]^**M**^
14	G	Zo	*Evandromyia*	*walkeri*	*L.* (*V*.) *bra* [24]^**M**^
15	S	Zo	*Expapillata*	*firmatoi*	*L.* (*L*.) *inf* [29]^**M**^
16	G	Ap	*Lutzomyia*	***almerio***	***L.* (*L*.) *inf*** [122]^**D&M**^; *L.* (*V*.) sp [122]^**M**^
17	G	Ap	*Lutzomyia*	***ayacuchensis***	*L.* (*L*.) *mex* [48]^**D**^; ***L.* (*V*.) *per*** [15]^**D**^
18	G	Ap	*Lutzomyia*	*cavenicola*	*L.* (*L*.) *inf* [20]^**M**^
19	G	Ap	*Lutzomyia*	***cruciat*** *a*	***L.* (*L*.) *mex*** [88]^**M**^
20	G	Ap	*Lutzomyia*	***cruzi***	***L.* (*L*.) *inf*** [25]^**M**^
21	G	Ap	*Lutzomyia*	*forattinii*	*L.* (*L*.) *inf* [25]^**M**^
22	G	Ap	*Lutzomyia*	*gaminarai*	*L.* (*L*.) *inf* [107]^**M**^; *L.* (*V*.) *bra* [107]^**M**^
23	G	Ap	*Lutzomyia*	***gomezi***	***L.* (*V*.) *bra*** [53]^**M**^; *L.* (*V*.) *nai* [109]^**M**^; *L.* (*V*.) *pan* [118]^**M**^; *L.* (*V*.) *sha* [27]^**D**^
24	G	Ap	*Lutzomyia*	*ischnacantha*	*L.* (*L*.) *inf* [105]^**M**^
25	G	Ap	*Lutzomyia*	***longipalpis***	*L.* (*L*.) *ama* [122]^**M**^; ***L.* (*L*.) *inf*** [13]^**D**^; *L.* (*V*.) *bra* [87]^**M**^
26	G	Ap	*Lutzomyia*	***peruensis***	***L.* (*V*.) *pe****r* [91]^**D**^
27	G	Ap	*Lutzomyia*	*renei*	*L.* (*V*.) *guy* [105]^**M**^
28	G	Ap	*Lutzomyia*	***tejadai***	*L****.* (*V*.) *bra/per*** [55]^**M**^
29	G	Ap	*Lutzomyia*	*tortura*	*L.* (*V*.) *nai* [57]^**D**^
30	G	Ap	*Lutzomyia*	*sherlocki*	*Lei* sp*.* [150]^**M**^
31	G	Zo	*Martinsmyia*	*minasensis*	*L.* (*V*.) *bra* [105]^**M**^; *L.* (*V*.) *guy* [105]^**M**^
32	G	Zo	*Micropygomyia*	*capixaba*	*L.* (*V*.) *bra* [105]^**M**^; *L.* (*V*.) *guy* [105]^**M**^
33	G	Zo	*Micropygomyia*	*cayennensis*	*L.* (*L*.) *mex* [98]^**M**^; *L.* (*V*.) *pan* [43]^**M**^
34	G	Zo	*Micropygomyia*	*ferreirana*	*L.* (*V*.) *bra* [110]^**M**^
35	G	Zo	*Micropygomyia*	*goiana*	*L.* (*V*.) *guy* [105]^**M**^
36	G	Zo	*Micropygomyia*	*peresi*	*L.* (*L*.) *inf* [105]^**M**^; *L.* (*V*.) *bra* [105]^**M**^
37	G	Zo	*Micropygomyia*	*quinquefer*	*L.* (*V*.) *bra* [87]^**M**^
38	G	Ap	*Migonemyia*	***migonei***	***L.* (*L*.) *inf*** [85]^**M**^; *L.* (*V*.) *bra* [6]^**D**^
39	G	Ap	*Nyssomyia*	*anduzei*	*L.* (*V*.) *guy* [28]^**D**^; *L.* (*V*.) *nai* [28]^**D**^
40	G	Ap	*Nyssomyia*	*antunesi*	*L.* (*V*.) *bra* [24]^**M**^; *L.* (*V*.) *nai* [69]^**M**^; *L.* (*V*.) *pan* [142]^**M**^
41	G	Ap	*Nyssomyia*	***intermedia***	*L.* (*L*.) *ama* [105]^**M**^; *L.* (*L*.) *inf* [25]^**M**^; ***L.* (*V*.) *bra*** [105]^-**M**^
42	G	Ap	*Nyssomyia*	***neivai***	*L.* (*L*.) *inf* [121]^**D**^; ***L.* (*V*.) *bra*** [94]^**M**^
43	G	Ap	*Nyssomyia*	***shawi***	***L.* (*V*.) *bra*** [38]^**M**^; *L.* (*V*.) *guy* [38]^**M**^; *L.* (*V*.) *nai* [126]^**M**^
44	G	Ap	*Nyssomyia*	***trapidoi***	*L.* (*L*.) *ama* [5]^**M**^; *L.* (*M*.) sp. [98]^**M**^; *L.* (*V*.) *nai* [8]^**M**^; ***L.* (*V*.) *pan*** [109]^**M**^
45	G	Ap	*Nyssomyia*	***umbratilis***	*L.* (*L*.) *ama* [127]^**M**^; *L.* (*V*.) *bra* [140]^**M**^; ***L.* (*V*.) *guy*** [28]^**D**^
46	G	Ap	*Nyssomyia*	***whitmani***	*L.* (*L*.) *ama* [132]^**M**^; *L.* (*L*.) *inf* [120]^**M**^; ***L.* (*V*.) *bra*** [6]^**D**^; ***L.* (*V*.) *guy*** [150]^**M**^; ***L.* (*V*.) *sha*** [27]^**D**^
47	G	Ap	*Nyssomyia*	***ylephilato*** *r*	***L.* (*L*.) *mex*** [95]^**D**^; *L.* (*V*.) *bra* [113]^**D**^ ***L.* (*V*.) *pan*** [77]^**D**^
48	G	Ap	*Nyssomyia*	*yuilli*	*L.* (*L*.) *ama* [127]^**M**^; *L.* (*M*.) sp*.* [98]^**M**^
49	G	Ap	*Pintomyia*	***evansi***	***L.* (*L*.) *inf*** [137]^**D**^; *L.* (*V*.) *bra* [96]^(*loc cit*)**M**^
50	G	Ap	*Pintomyia*	*fischeri*	*L* (*L*.) *inf* [37]^**D**^, [107]^**M**^; *L.* (*V*.) *bra* [110]^**M**^
51	G	Ap	*Pintomyia*	***longiflocosa***	*L****.* (*V*.) *guy*** [35]^**M**^
52	G	Ap	*Pintomyia*	*monticola*	*L.* (*L*.) *inf* [29]^**M**^
53	G	Ap	*Pintomyia*	***nuneztovari***	*L.* (*L*.) *ama* [9]^**M**^; ***L.* (*V*.) *bra*** [9]^**M**^; *L.* (*V*.) *lai* [9]^**M**^
54	G	Ap	*Pintomyia*	***ovallesi***	*L.* (*L*.) *mex* [53]^**M**^; *L.* (*L*.) *inf* [117]^**M**^; ***L.* (*V*.) *bra*** [114]^**D**^; *L.* (*V*.) *pan* [117]^**M**^
55	G	Ap	*Pintomyia*	*pia*	*L.* (*V*.) *pan* [71]^**M**^
56	G	Ap	*Pintomyia*	***spinicrassa***	*L.* (*L*.) *ama* [117]^**M**^; ***L.* (*V*.) *bra*** [149]^**D**^
57	G	Ap	*Pintomyia*	*townsendi*	*L.* (*L*.) *ama* [52]^**M**^
58	G	Ap	*Pintomyia*	***verrucarum***	***L.* (*V*.) *per*** [23]^**D**^
59	G	Zo	*Pressatia*	*dysponeta*	*L.* (*V*.) *nai* [98]^**M**^
60	G	Zo	*Pressatia*	*tricantha*	*L.* (*M*.) sp. [98]^**M**^
61	G	Ac	*Psathyromyia*	*aragaoi*	*L.* (*V*.) *bra* [87]^**M**^
62	G	Ac	*Psathyromyia*	*barrettoi*	*L.* (*L*.) *ama* [5]^**M**^
63	G	Ac	*Psathyromyia*	*naftalekatzi*	*Lei* sp. [47]^**M**^
64	G	Ac	*Psathyromyia*	*shannoni*	*L.* (*L*.) *mex* [88]^**M**^
65	G	Ac	*Psathyromyia*	***cratifer***	***L.* (*L*.) *mex*** [81]^**M**^
66	G	Ap	*Psychodopygus*	*amazonensis*	*L.* (*V*.) *bra* [108]^**M**^; *L.* (*V*.) *nai* [46]^**M**^; *Lei* [64]^**D**^
67	G	Ap	*Psychodopygus*	*ayrozai*	*L.* (*V*.) *bra* [24]^**M**^; *L.* (*V*.) *nai* [4]^**D**^
68	G	Ap	*Psychodopygus*	*carrerai*	*L.* (*V*.) *bra* [45]^**D**^; *L.* (*V*.) *nai* [98]^**M**^; *L.* (*M*.) sp*.* [98]^**M**^
69	G	Ap	*Psychodopygus*	*chagasi*	*L.* (*V*.) *bra* [108]^**M**^
70	G	Ap	*Psychodopygus*	*claustrei*	*L.* (*V*.) *bra* [87]^**M**^
71	G	Ap	*Psychodopygus*	***complexus***	***L.* (*V*.) *bra*** [26]^**D**^
72	G	Ap	*Psychodopygus*	***davisi***	*L.* (*L*.) *ama* [108]^**M**^, ***L.* (*V*.) *bra*** [39]^**D**^; ***L.* (*V*.) *na****i* [39]^**D**^
73	G	Ap	*Psychodopygus*	*hirsutus*	*L.* (*V*.) *bra* [22]^**M**^; *L.* (*V*.) *nai* [27]^**D**^
74	G	Ap	*Psychodopygus*	*llanosmartinsi*	*L.* (*V*.) *bra* [24]^**M**^
75			*Psychodopygus*	*lloydi*	*L.* (*L*.) *inf* [66]^**M**^, *L.* (*V*.) *bra* [132]^**M**^
76	G	Ap	*Psychodopygus*	*maripaensis*	*L.* (*V*.) *nai* [27]^**D**^
77	G	Ap	*Psychodopygus*	*panamensis*	*L.* (*L*.) *ama* [71]^**M**^; *L.* (*L*.) *mex* [89]^**M**^; *L.* (*L*.) *maj* [98]^**M**^; *L.* (*V*.) *bra* [113]^**D**^; *L.* (*V*.) *nai* [98]^**M**^; *L.* (*V*.) *pan* [118]^**M**^; *L.* (*M*.) sp. [98]^**M**^
78	G	Ap	*Psychodopygus*	*paraensis*	*Lei* sp. [64]^**D**^; *L.* (*V*.) *nai* [4]^**D**^
79	G	Ap	*Psychodopygus*	*squamiventris*	*L.* (*V*.) *bra* [45]^**D**^; *L.* (*V*.) *nai* [45]^**D**^
80	G	Ap	*Psychodopygus*	***wellcome*** *i*	***L.* (*V*.) *bra*** [64]^**D**^
81	G	Ap	*Psychodopygus*	***yucumensi*** *s*	***L.* (*V*.) *bra*** [67]^**D**^
82	S	Zo	*Sciopemyia*	*sordellii*	*L.* (*L*.) sp. [20]^**M**^; *L.* (*V*.) *nai* [126]^**M**^
83	S	Ac	*Trichophoromyia*	*auraensis*	*L.* (*V*.) *bra* [133]^**M**^; *L.* (*V*.) *guy* [133]^**M**^; *L.* (*V*.) *lai* [139]^**M**^
84	S	Ac	*Trichophoromyia*	*brachipyga*	*L.* (*V*.) *lai* [138]^**D**^
85	S	Ac	*Trichophoromyia*	*ininii*	*L.* (*V*.) *bra* [140]^**M**^
86	S	Ac	*Trichophoromyia*	*reburra*	*L.* (*L*.) *ama* [5]^**M**^
87	S	Ac	*Trichophoromyia*	*ruifreitasi*	*L.* (*L*.) *bra* [133]^**M**^; *L.* (*V*.) *guy* [133]^**M**^
88	S	Ac	*Trichophoromyia*	*triramula*	*L.* (*V*.) *nai* [109]^**M**^
89	S	Ac	*Trichophoromyia*	***ubiquitalis***	*L.* (*L*.) *ama* [127]^**M**^; ***L.* (*V*.) *lai*** [129]^**D**^
90	S	Zo	*Viannamyia*	***tuberculata***	***L.* (*V*.) *uti*** [12]^**D**^
91	G	Ap	*Warileya*	*rotundipennis*	*L.* (*V*.) sp. [83]^**M**^
			18 genera	91 species	157 separate infections

Legend: Ha = Habit; Fe = Feeding preference; G = Generalist; S = Specialist; Ac = Anthroaccidentalist; Ap = Anthropportunist; Ph = Phytophagous; Zo = Zoophilic; *L.* (*L.*) *ama = Leishmania* (*Leishmania*) *amazonensis*; *L.* (*L*.) *inf = L.* (*L*.) *infantum*; *L.* (*L*.) *maj = L.* (*L.*) *major*-like; *L.* (*L*.) *mex* = *L.* (*L*.) *mexicana*; *L.* (*V*.) *bra* = *L.* (*Viannia*) *braziliensis*; *L.* (*V*.) guy = *L.* (*V*.) *guyanensis*; *L.* (*V*.) *lai = L.* (*V*.) *lainsoni*; *L.* (*V*.) *nai* = *L.* (*V*.) *naiffi*; *L.* (*V*.) *pan = L.* (*V*.) *panamensis*; *L.* (*V*.) *sha* = *L.* (*V*.) *shawi*; *L.* (*V*.) *uti = L.* (*V*.) *utingensis*; *Lei* sp*.= Leishmania* species; *L.* (*L*.) sp. *= L.* (*Leishmania*) species; *L.* (*M*.) sp. = *L.* (*Mundinia*) species; *L.* (*V*.) sp. = *L.* (*Viannia*) species. Detection Method: D = Dissection live parasite, M = Molecular extracted DNA; Bold species and its acronym indicates a proven vector of a *Leishmania* species.

The species mentioned below have been found to be infected with *Leishmania* during American cutaneous leishmaniasis (ACL) and American visceral leishmaniasis (AVL) epidemiological studies. Based on the available data, they are categorized as generalist or specialists and then anthropportunists, anthroaccidentalists, or zoophilic. The major difference between the last two is the absence of evidence indicating humans as a source of food.

## Genus *Bichromomyia* and *Leishmania* (*Leishmania*) *mexicana* complex parasite vectors

Members of this genus are found throughout Central and South America and transmit parasites of the *L.* (*L.*) *mexicana* clade [11, 45, 62], but evidence of their anthropophily under certain situations is based more on the fact that these parasites are found in humans. In Yucatan state, Mexico, *Bichromomyia olmeca* is considered to be the vector, but the low number of cases suggests that it is anthroaccidentalist. Other species may transmit *L*. (*L.*) *mexicana*, as human infections occur outside *Bi. olmeca*’s distribution. It has been suggested [44] that *Lutzomyia cruciata* and *Psathyromyia shannoni* would be the most suitable candidates as both are distributed broadly and have been found to be infected with *L*. (*L*.) *mexicana*. All three species have been considered as being “highly anthropophilic” [18], but the reasons for this statement are not given. This could be because they were the dominant species in Shannon traps [88], but it does not mean that they would feed on people. A series of chemicals such as carbon dioxide and semiochemicals [14] attract sand flies. Nonetheless, there is a lack of data on the molecules emitted by vertebrates, sand flies, and plants that affect phlebotomine behavior. If these molecular signatures were compatible, the females would feed on either plants or animals. A species may be attracted, but if its chemoreceptors were not tuned to the host’s semiochemicals, it would not feed. This is very important in host specificity. *Leishmania* (*L*.) *amazonensis*’s vector is *Bi. flaviscutellata* and given a choice of hosts, the majority feed on rodents. That said, the species has been collected from humans in small numbers [124], while large numbers were collected from rodents. However, in ecotopes where the populations were high, the human-biting index was 1.25 flies per hour [125]. The available evidence indicates that *Bi. flaviscutellata* can be considered an anthroaccidentalist species [17], but its biting index is dependent on the population density in specific biomes. *Bichromomyia* species are strongly attracted to rodents and given their importance as reservoirs [74] of *L.* (*Viannia*) *braziliensis*, it is very surprising that this parasite has only been recorded in this genus on one occasion [140]. It has been shown experimentally that *Bi. flaviscutellata* does not support the development of *L.* (*Viannia*) species [30, 101]. The single recorded infection [140] was a molecular identification and was probably a dead-end infection that would subsequently be excreted. This is a good example of how laboratory experimental infections can help to interpret field results.

In other regions such as Texas, United States the vector of *L*. (*L*.) *mexicana* is considered to be a rodent loving species, *Dampfomyia anthophora* [78], and human cases are extremely rare. It has been suggested that other species such as *Lutzomyia diabolica* or *Psathyromyia shannoni* may be responsible for the human infections. There are no records of this species seeking humans as a food source, so it has been considered zoophilic.

## The genus *Evandromyia*

Species of this genus are not considered to feed on humans, but have been found to be infected with *L.* (*V.*) *braziliensis* [3, 20, 112] and *L.* (*L.*) *infantum* [19, 20, 121]. Their importance in leishmanial enzootic cycles has yet to be determined. They are generally taken in light traps. An infection of *L*. (*V.*) *braziliensis* has been recorded on one occasion [24] in *Ev. infraspinosa.* This species has been collected in small numbers in Shannon traps, while 97% were captured in light traps [27]. It is not considered to be attracted to humans [17]. An isolate from *Ev. infraspinosa* captured in Rondônia, Brazil was identified [34] as a trypanosome of anurans. There are records of *L.* (*L.*) *infantum* and *L.* (*V.*) *braziliensis* [131] in several *Evandromyia* species that have been captured in light traps. The available evidence strongly suggests that *Evandromyia* species are zoophilic generalists, indicating that they could play a role in enzootic but not zoonotic cycles. Attempts to infect laboratory reared *Ev. carmolinoi* with different *Leishmania* have failed [115]. Alternatively, the recorded infections in *Evandromyia* species might be ones that die out. These questions could be answered by dissections of wild caught flies and experimental infections in laboratory reared females.

## The genus *Lutzomyia*

Species of this subgenus are vectors of cutaneous and visceral leishmaniasis-causing parasites. *Lutzomyia longipalpis* is a complex of genetically distinct species [143, 146] responsible for the transmission of *L.* (*L*) *infantum* throughout Central and South America. It was first associated with visceral leishmaniasis in 1934 by Penna [90], and similar observations by other Latin American workers cemented it as the vector of American visceral leishmaniasis. However, it was only shown to experimentally transmit *L.* (*L*) *infantum* in 1977 [65]. Finally, isolates from naturally infected flies were shown to have the same enzymatic profiles in 1986 [13] as those isolated from humans. The degree of attraction to humans was uncertain in Amazonia, but studies have shown that in the peri-domestic environment, attractiveness for this species is largely related to the host’s size and accessibility [97]. It appears to feed on what is most readily available, in many cases large animals such as pigs and cows, but in houses it feeds predominantly on humans [84], indicating that it is an anthropportunist. However, given the choice, it is attracted preferentially to dogs and chickens instead of humans [17]. It has been found to be infected with *L.* (*L*) *amazonensis* [122] and *L.* (*V.*) *braziliensis* [87], but its preference for the known reservoirs of these two species suggests that it may play a minimal role, if any, in their transmission.

In some Andean regions of Ecuador and Peru, *Lu. ayacuchensis* is the dominant species collected from humans, where it has been captured in large numbers [41]. In both countries [48, 58], it has been found infected with a parasite that was identified as belonging to the *L.* (*L*) *mexicana* complex. Epidemiological studies have shown that this species is the vector [50] of Andean cutaneous leishmaniasis in Ecuador’s Chimborazo province. It has also been found to be infected with *L.* (*V.*) *peruensis* in Ayacucho, Peru [15] and should be considered an anthropportunist generalist.

In Mato Grosso do Sul, Brazil *Lu. almerioi* and *Lu. forattinii* are closely related to *Lu. longipalpis* and are a “highly anthropophilic species” [122]. Both have been found to be infected with *L.* (*L*) *infantum* as has *Lu. cruzi* [25], and are considered AVL vectors in Mato Grosso do Sul, indicating that these three species are anthropportunists.

*Lutzomyia cruciata* is an anthropportunistic species, as shown by collection from humans [31, 104] and in Shannon traps [88] in Mexico. It has been found to be infected with *L.* (*L*) *mexicana* in Mexico’s Yucatan peninsula [88, 116]. This sand fly occurs in southern Texas and Central America, and it has been reported as far south as Panama [36].

*Lutzomyia gaminarai* was described from Uruguay and also occurs in Paraguay, as well as in the southern Brazilian states of Rio Grande do Sul and Parana [36]. It was the dominant species captured in light traps set in anthropogenic environments during an outbreak of visceral leishmaniasis in Porto Alegre, Brazil and was found to be infected with both *L.* (*L*) *infantum* and *L.* (*V.*) *braziliensis* [107].

*Lutzomyia gomezi* has a very extensive geographical distribution throughout Central and South America [36] and can be considered to be anthropportunistic [31, 41]. In Amazonia, its attraction to humans was like that of *Ny. whitmani* [17]. It has been found to be infected with *L.* (*V.*) *braziliensis* [111], *L.* (*V.*) *naiffi* [8], *L.* (*V.*) *panamensis* [118], *L.* (*V.*) *shawi* [27], and *Endotrypanum* [59]. The fact that it is infected with so many different parasites fully supports the view that it is a generalist. Given its parasitological background, this species is clearly an anthropportunist. Although it occurs in many countries, its density varies, which impacts its epidemiological importance as an ACL vector.

*Lutzomyia hartmanni* has been recorded as being attracted to humans in Ecuador [41], and has been found to be infected with *Endotrypanum* species but not any *Leishmania* species. Sloths are hosts to both parasites, leading to the question of why no *Leishmania* infections have been recorded in this species. It could be refractory to *Leishmania* infection, but this seems unlikely given leishmanial susceptibility of *Lutzomyia* species. To date, the available data indicate that this is an anthropportunistic species of no leishmanial vectorial importance. An unknown *Leishmania* has been recorded in *Lu. sherlocki* in the Peruvian Amazon [150]. This species occurs in Bolivia, Brazil, Colombia, Ecuador, and Peru [36].

*Lutzomyia peruensis* occurs in the Andean highlands of Bolivia and Peru and has been found to be infected [91] with *L.* (*V*.) *peruviana* in a number of Peruvian localities above 2,000 m above sea level [49]. It is an anthropportunistic species [91], is distributed on the western side of the Andes [82], and is the principal vector of uta due to its endophilic habit [144].

*Lutzomyia tortura* is a species with limited geographical distribution in Bolivia, Ecuador, and Colombia [36] and, so far, the evidence indicates that it is an anthropportunist species in Ecuador [41]. In this country, it has been found to be infected with *L.* (*V.*) *naiffi* [57] and was considered the vector of this parasite to humans.

*Lutzomyia tejadai* was captured in CDC and Shannon traps in and around houses in a Peruvian endemic ACL focus situated approximately 3,000 m above sea level. A single infection was found in one of these sand flies that was identified as a hybrid *L.* (*V.*) *braziliensis*/*L.* (*V.*) *peruviana* [55]. Human infections of this hybrid have been reported in the area, strongly supporting *Lu. tejadai*’s vectorial role and it being considered an anthropportunist.

## The genus *Migonemyia*

The only species of this genus associated with leishmanial parasites is *Mi. migonei.* It is found in most Brazilian States as well as Argentina, Bolivia, Colombia, Venezuela, and Peru [36]. It is attracted to humans and has been found to be infected with both *L.* (*V.*) *braziliensis* [7] and *L.* (*L.*) *infantum* [85]. Interest in this species’ epidemiological importance is linked to it being anthropportunistic and being found in anthropogenic environments.

## The genus *Nyssomyia*

Species of this genus are among the most important ACL vectors throughout the Americas and are found in both sylvatic and anthropogenic environments. Among the 21 *Nyssomyia* species, *Leishmania* infections have been found in ten (*Ny. anduzei*, *Ny. antunesi*, *Ny. intermedia*, *Ny. neivai*, *Ny. shawi*, *Ny. trapidoi*, *Ny. umbratilis*, *Ny. whitmani*, *Ny. ylephilator*, and *Ny. yuilli*). For some, there are records of individual infections with up to three *Leishmania* species. The available evidence of their biology and infections indicates that these ten species are anthropportunistic generalists.

*Leishmania* (*V.*) *guyanensis* have been recorded in *Ny. anduzei* and *Ny. umbratilis* and both are anthropportunists [28, 103]. Additionally, *L.* (*V.*) *naiffi* has been found in *Ny. anduzei* [28] and *L.* (*L.*) *amazonensis* in [127] *Ny. umbratilis*. This suggests that besides being the vectors of zoonotic *L.* (*V.*) *guyanensis*, they may play a vectorial role for other *Leishmania*. *Nyssomyia antunesi* occurs in more open areas [141] and has been found to be infected with *L* (*V*.) *braziliensis* [24], *L* (*V*.) *naiffi* [126], and *L* (*V*.) *panamensis* [142]. Its presence in houses makes it a potential anthropportunistic generalist ACL vector.

*Nyssomyia intermedia* and *Ny*. *neivai* are closely related species [2, 86] and both occur in anthropogenic environments. They are accepted as ACL vectors and are found in the drier eastern regions of the South American continent from Rio Grande do Norte to Argentina and Paraguay [36]. It is considered that older southerly records of *Ny. intermedia* refer to *Ny*. *neivai* but in São Paulo state they are sympatric. There are records of *L.* (*V.*) *braziliensis* [72, 94, 105, 110] in both species and *Ny. intermedia* blood meal analyses indicate that it is an eclectic feeder [10], showing that it is an anthropportunistic generalist. In a rural area of Minas Gerais, *Ny. intermedia* has been found to be infected [105] with *L.* (*V.*) *braziliensis*, *L.* (*L.*) *infantum*, and *L.* (*L.*) *amazonensis.* The earliest 1922 records [2] of *Ny. intermedia* presence in the homes of infected people indicated its anthropophily, and subsequent studies [130] have confirmed this.

The first indication of *Ny*. *shawi*’s anthropophily was in Bolivia [38] where it was found inside human dwellings and collected in Shannon traps in large numbers. In this study where *L.* (*V.*) *braziliensis* ACL was endemic, it was found to be infected with *L.* (*V.*) *braziliensis* and *L.* (*V.*) *guyanensis*, strongly indicating that it is an anthropportunistic generalist.

*Nyssomyia trapidoi* is a Central American species that is also found in Colombia and Ecuador [36]. It feeds on humans [32] and occurs in anthropogenic environments [80]. It has been found to be infected with *L.* (*V.*) *panamensis* [68, 77] and more recently with *L.* (*V.*) *naiffi* [8, 109] and *Endotrypanum* [41]. With changing environments, this species is responsible for *L.* (*V.*) *panamensis* [109] ACL. It has been shown to feed on a wide range of wild mammals, particularly primates [135], suggesting that it is an anthropportunistic generalist and an important ACL vector.

*Nyssomyia whitmani* has a long history of anthropophily [40] and is considered to be an important vector of *L.* (*V.*) *braziliensis* ACL throughout its extensive distribution in practically all Brazilian States, as well as in Argentina, Bolivia, Paraguay, Surinam, and the Guyanas [36]. In some areas, in overlaps with another important ACL vector, *Ny. intermedia*. Molecular studies [99] have shown distinct geographical populations and that there are some behavioral differences [17]. In different regions, it has been found to be infected with *L.* (*V.*) *braziliensis* [6], *L.* (*V.*) *guyanensis* [28]*, L.* (*V.*) *shawi* [61]*, L.* (*L.*) *amazonensis* [132], and *L.* (*L.*) *infantum* [120]. The evidence clearly shows that this is an anthropportunistic species and an important ACL vector of the first two parasite species. However, its participation in a cycle involving visceral parasite is uncertain.

*Nyssomyia ylephilator* is a Central American species that has been found to bite humans predominantly in forest environments, but also inside houses [32] and can be considered an anthropportunistic vector. It has been found to be infected with *L.* (*V.*) *braziliensis* [113], *L.* (*V.*) panamensis [77], and *L.* (*L.*) *mexicana* [95]*.* In Guatemala it was considered an *L.* (*V.*) *braziliensis* ACL vector together with *Pintomyia ovallesi* and *Psychodopygus panamensis* [113]*.*

In the Amazon region of Ecuador, *Ny. yuilli yuilli* is anthropophilic because it is found in domestic settings. It has been found to be infected with *L.* (*L.*) *amazonensis* in the Brazilian Amazon [127], *L.* (*V.*) *panamensis* [118] in a domestic location in Colombia, and *Endotrypanum* sp., [41] and a possible *L.* (*Mundinia*) sp., [98] in Ecuador. This variety of parasites reflects eclectic feeding, but so far it has not been implicated as an ACL vector. Studies of vertical distribution showed this species to be more common in canopy catches [69]. Given these different characters, this species appears potentially to be an anthroaccidental generalist.

## The genus *Pintomyia*

Species of this genus feed on humans, are found in anthropogenic biomes, and are considered to be important vectors of *L.* (*Viannia*) and *L.* (*Leishmania*) parasites. Infections of *L.* (*L.*) *infantum* have been found in *Pi. evansi* in Colombia [137]. This species extends from Mexico throughout Central America down into Peru and Venezuela [36]. In Honduras, it is considered a sympatric vector of VL, preferentially feeding on dogs and pigs [79]. A less well-known species, *Pi*. *longiflocosa* has so far only been found in Colombia [36]. *Pintomyia fischeri* has a long history of being a suspected leishmaniasis vector in Brazil. It has been found to be infected with *L.* (*L.*) *braziliensis* [110] and *L.* (*L.*) *infantum* [37]. In an ACL epidemic in Chaparral, Colombia [35] it was the dominant species, highly attracted to humans and endophilic. Infections with *L.* (*V*.) *guyanensis* were identified in patients and *Pi*. *longiflocosa*. An outbreak of *L* (*L*.) *amazonensis* ACL occurred in Inquisivi Province, La Paz [76] and infections with this parasite were later found in *Pi. nuneztovari* [75]. Infections with *L.* (*V.*) *braziliensis* [136] and *L.* (*V.*) *lainsoni* [9] have also been found in this same sand fly species. An infection with *L.* (*V.*) *panamensis* has been recorded [71] in *Pi. pia* captured in Colombian montane forest. The parasitological and epidemiological evidence indicates that the six species mentioned in this paragraph are anthropportunistic generalists.

*Pintomyia ovallesi* has an extensive geographical distribution that stretches from Mexico to Colombia, and then easterly across Venezuela to Trinidad [36]. It is attracted to humans in Central America [114] and in Venezuela, where it is endophilic [33]. It has been found to be infected with *L.* (*L*.) *infantum* [117], *L.* (*L*.) *mexicana* [53], *L.* (*V*.) *braziliensis* [114], and *L* (*V*.) *panamensis* [117], indicating that it is an anthropportunistic generalist. Given its endophilic habits, it is considered an *L.* (*V*.) *braziliensis* ACL vector and has potential vectorial roles for other parasites. *Pintomyia robusta* was shown to be attracted to humans, but so far has only been found to be infected with *Endotrypanum* [41], and because of this, it is not included in Table 1*.*

*Pintomyia spinicrassa* has a limited distribution in Colombia and Venezuela [36], is anthrophilic, and has been collected from humans in Colombia [1, 149]. It has been found to be infected with *L.* (*V*.) *braziliensis* in the mountainous area of Arboledas [149], in the Santander region with *L.* (*L*.) *amazonensis*, *L.* (*V*.) *braziliensis*, and *L.* (*V*.) *panamensis* [117], and in the Venezuelan Andean region with *L.* (*V*.) *braziliensis* [92]. It is considered one of the main *L.* (*V*.) *braziliensis* ACL vectors in these three regions and is characteristic of an anthropportunistic generalist.

Three *Pintomyia* species, *Pi. columbiana, Pi. townsendi*, and *Pi. verrucarum* have limited distributions in Peru, Columbia, and Venezuela [36]. All are considered as occurring in anthropogenic biomes and are attracted to humans [23, 145]. *Leishmania* (*V*.) *peruviana* has been recorded [23] in *Pi. verrucarum* and it was shown to transmit the same parasite experimentally. *Leishmania* (*L*.) *amazonensis* has been recorded in *Pi. townsendi* [52]. The available evidence indicates that these three species are anthropportunistic generalists.

## The genus *Psathyromyia*

Of the species belonging to this genus, *Psa. shannoni* has the greatest geographical distribution from the United States down to southern Brazil [36]. In many situations, it is collected in small numbers [41] and in Amazonia, ten different species were captured in CDCs, but only one female in a Shannon trap [28]. However, in Mexican villages where ACL was endemic, it was the only species found to be infected with *L.* (*L*.) *mexicana* and was attracted to humans in Shannon traps [88]. *Leishmania* (*V*.) *braziliensis* has been found in *Psa. aragoi* [87], *L.* (*L*.) *amazonensis* in *Psa. barrettoi majuscule* [5]*, L.* (*L*.) *mexicana* in *Psa. cratifer* [81], and an unidentified *Leishmania* in *Psa. naftalekatzi* [47]. In another endemic Mexican ACL region, *Psa. cratifer* was the dominant species collected in Shannon traps, but not in CDC traps [81]. The overall evidence indicates that *Psathyromyia* species are anthroaccidental generalists, but in specific environments that favor large populations, they may be the main species attracted to humans and therefore ACL vectors.

## The genus *Psychodopygus*

The greatest number of *Psychodopygus* species is found in Amazonia, but few have been recorded in the Atlantic forest and Central America. To date, *L.* (*Viannia*) infections have been recorded in 13 *Psychodopygus* species. Of these, *L.* (*V.*) *braziliensis* was recorded in ten species and *L.* (*V.*) *naiffi* in seven species. There is conclusive evidence [64] that *Ps. wellcomei* bites humans in areas such as the Carajás mountains of Pará State, Brazil. There may be five times more *Ps. wellcomei* than *Ps. davisi* collected from humans. However, in regions such as Rondônia, where *Ps. wellcomei* does not occur, *Ps. davisi* was dominant in Shannon traps [39] and has been found to be infected with *L.* (*V.*) *braziliensis.* In the Paragominus region of Pará State, Brazil *Ps. complexus* was infected with *L.* (*V.*) *braziliensis* and attracted to humans [26]. This is a sympatric morphospecies with *Ps. wellcomei* and their females can only be differentiated by molecular methods [102].

The above are examples of the changing dominance of *Psychodopygus* species in different regions, where all are considered ACL vectors. There are records of 12 different *Psychodopygus* species in Rondônia [39] and 11 in the Tapajós FLONA (FLOresta NAcional), Pará State [27]. In both regions, *Ps. davisi* was dominant and there was a noticeable absence of *Ps. complexus*/*Ps. wellcomei. Leishmania* (*L*.) *amazonensis* [108] as well as *L.* (*V.*) *braziliensis* and *L.* (*V.*) *naiffi* [39] have been record in *Ps. davisi* from Rondônia. This suggests that the same species transmit the latter two ACL parasites. Parasites of the subgenus *L.* (*Leishmania*) are rarely found in *Psychodopygus* species, suggesting the existence of biological or environmental barriers preventing them from being infected and of being involved in their transmission.

*Leishmania* (*V.*) *braziliensis* has been recorded in *Ps. amazonensis* [108], *Ps. ayrozai* [24], *Ps. carrerai* [45]*, Ps. chagasi* [108], *Ps. clausterei* [87], *Ps. hirsutus* [22], *Ps. llanosmartinsi* [24], *Ps. lloydi* [132], *Ps. panamensis* [113], *Ps. squamiventris* [45], and *Ps. yucumensis* [67]. *Leishmania* (*V.*) *naiffi* has been recorded in *Ps. amazonensis* [46], *Ps. ayrozai* [4], *Ps. carrerai* [98], *Ps. hirsutus* [27], *Ps. maripaensis* [28], *Ps. paraensis* [4], and *Ps. squamiventris* [45]*.* Seven leishmanial parasites have been recorded in *Ps. panamensis*: *L.* (*L.*) *amazonensis* [71], *L.* (*L.*) *mexicana* [89], *L.* (*V.*) *braziliensis* [113], *L.* (*V.*) *naiffi* [109], *L.* (*V.*) *panamensis* [118], *L.* (*Mundinia*) sp., [98], and a *major-*like parasite [98]. Only once has *L.* (*L.*) *infantum* been found in a *Psychodopygus* species, *Ps. lloydi* [66], reflecting the predominantly sylvatic habits of the species in this genus. The variety of parasites reflects captures in different regions and biomes, but reflects the generalist habits of these species given the wide variety of animals that host them.

There are *bona fide* records that *Ps. amazonensis*, *Ps. davisi*, *Ps. carrerai*, and *Ps. paraensis* bite humans [147]. In the original description, *Ps. panamensis* was recorded as biting humans [31] as well as being taken in human landing captures [41]. Taken together, these records, along with the different associated Leishmaniinae infections, strongly indicate that *Psychodopygus* species are anthropportunistic generalists.

## The genus *Trichophoromyia*

*Trichophoromyia ubiquitalis* is the vector [129] of *L.* (*V*.) *lainsoni.* Records of flies do not appear in captures taken from human bait [147], but it is captured in light traps [28, 39, 129, 138] and in smaller numbers in Shannon traps [28]. However, on a single occasion, one female was captured biting a man [63] and there are human infections of *L*. (*V*.) *lainsoni* [128]. From this, it appears that under exceptional conditions, *Th. ubiquitalis* will bite humans, and as such, it is an anthropportunistic ACL vector. In the Belém metropolitan region, ten *L.* (*V*.) *lainsoni* ACL cases were seen over a 23-year period that represented 0.4 cases per year [42]. In an entomological survey in the same region, 23% of the phlebotomines captured in light traps were *Th. ubiquitalis.* This is an example of how an anthroaccidentalist can be responsible for a constant low level of ACL transmission. There is a single record of *L.* (*L.*) *amazonensis* in this same sand fly captured in Amazonas [127].

*Leishmania* (*V.*) *naiffi* is recorded in *Th. triramula* captured in Panama [109]. Infections with *L.* (*V.*) *braziliensis* have been found in *Th. aurensis* from Acre, Brazil [24, 133], *L.* (*V.*) *guyanensis* [133] and *L.* (*V.*) *lainsoni* [139] captured in Madre de Dios, Peru [139]. In both regions, *L.* (*V.*) *lainsoni* has been found in humans. *Leishmania* (*V.*) *braziliensis* has been recorded in *Th. reburra* from Ecuador [5] and *Th. ininii* from Brazil [140]. Infections with *L.* (*V.*) *braziliensis* and *L.* (*V.*) *guyanensis* have been found in *Th. ruifreitasi* captured in Acre, Brazil [133]. A recent review [119] of the vectorial importance of this sand fly genus concluded that there was increasing evidence of their putative role as vectors. I agree with this and consider that the available evidence suggests that *Trichophoromyia* species are anthroaccidental specialist ACL vectors. In Peruvian regions where over 60% of the sand flies were *Th. aurensis* [139], its ACL vectorial potential is greatly increased.

There are 57 named *Trichophoromyia* species [36]. So far only one, *Th. triramula*, has been found in Central America and they are absent in the southern regions of South America, including southern Brazil. The coincidental geographical distribution of *Th. ubiquitalis* [148] and *L.* (*V.*) *lainsoni*, but not with that of the paca (*Agouti paca*), its proven reservoir, strongly suggests a specific vector/parasite relationship between *Trichophoromyia* species and *L.* (*V.*) *lainsoni*.

## Genus *Warileya*

Eight *Warileya* species have been described. In an endemic *L.* (*V.*) *panamensis* ACL locality of Colombia, *W. rotundipennis* represented 98% of the sand flies captured inside houses. It was found to be infected with a *L.* (*Viannia*) species and blood meal analysis was positive for human, sloth, cow, and marsupial blood [83]. Of the blood meals, 95% were human. This is not surprising as catches were performed with CDCs inside houses. These data indicate that *W. rotundipennis* is an anthropportunistic generalist. *Warileya* species can be found in different biomes ranging from caves and crevices to tree trunks. This is the first time that a species of this genus has been found inside houses, suggesting that some new environmental event may have occurred.

## Zoophilic and nectar-feeding genera

*Leishmania* have been recorded in species of the following genera: *Brumptomyia* [98]*, Expapillata* [29], *Martinsmyia* [105], *Micropygomyia* [43, 87, 98, 105, 106, 110], *Pressatia* [98, 109], *Sciopemyia* [20, 126], and *Viannamyia* [12]. It is unknown whether these represent chance infections due to a fortuitous encounter with an infected host, are involved in the maintenance of an enzootic cycle, or are transient infections that do not lead to transmission. All of the recorded parasites, except *L.* (*V.*) *utingensis*, found in *L.* (*V.*) *tuberculata*, have been found in humans. The identification of a *L.* (*Mundinia*) species in *Br. leopoldoi* is surprising as, so far, *Brumptomyia* species are considered nectar feeders. Also, the vectors of *L.* (*Mundinia*) species are midges. Could these parasites be trypanosomatids of plants being transmitted by phlebotomines?

The feeding habits of the species classified as zoophilic are poorly understood, and further ecological studies are needed to clarify their role in transmission and host interactions. Species of these genera are considered to be nectar feeders or hematophagous.

## Discussion

Given phlebotomine sand flies’ antiquity and their feral life cycles, it is unlikely that their human seeking and anthropogenic biomes (anthropophily) are the result of evolutionary selection. This is more likely related to pre-existing behavioral traits. However, insects adapt rapidly to selective pressures, such as the dominance of melanisms of the peppered moth [21] and insecticide resistance [70]. There is evidence that sand fly populations that have been exposed to insecticides for many years become resistant [54]. As a result, it is possible that there could be behavioral selection for anthropogenic biomes that would bring them into greater contact with humans. One possibility would be gene flow or introgression between species found in the same biome that could potentially contribute to adaptation and evolution. Introgression has been found between two anthropportunistic species, *Ny. intermedia* and *Ny. whitmani* [73]. The former is well adapted to anthropogenic biomes. In areas where they are sympatric, *Ny. whitmani* lineages thrive in peri-domestic environments, but where they are not sympatric, they do not. This suggests that gene flow may have been responsible for this and other behavioral changes of *Ny*. *whitmani*.

The available evidence indicates that species that bite humans are generalists that evolved in environments where they feed opportunistically on a wide range of animals. For instance, in Colombia [43], *Lu. gomezi* was found to have fed on chickens, pigs, dogs and humans, confirming its generalist and anthropportunistic habits. Epidemiologically important anthropportunistic species belong to the genera *Lutzomyia*, *Nyssomyia*, *Pintomyia*, and *Psychodopygus*. However, there are biological distinctions between them. All occur in natural biomes, but so far, *Psychodopygu*s species have not adapted to anthropogenic environments as have some species of the other three genera. Such behavior increases their chance of feeding on humans and consequently being the main ACL vectors. It has been shown that *Ny. whitmani* preferentially returns to a site [16] where a particular food source is located. This would clearly favor contact with that host. In southern Brazil, *Ny. whitmani* is found in greater numbers in anthropogenic sites [134], which enhances its contact with humans and chances of feeding on them.

To date, *Ny. whitmani* has been shown to consist of at least three mitochondrial lineages [99], one in Amazonia and two in eastern Brazil. Studies on their attractiveness [17] indicated that the most southeastern lineage had a greater preference for humans than the other two and that the greatest numbers of individuals in the southern lineage occurred in peri-domestic environments. Previously Amazonian *Ny. whitmani* populations were recorded as not being attracted to human-based ground level catches in forested regions [60]. A possible explanation for these conflicting conclusions is that population densities of this species are lower at ground level and higher in the canopy. Additionally, *Ny. whitmani* was considered to be the vector of *L.* (V.) *shawi* and an outbreak of this parasite occurred in southeast Amazonia [123]. Subsequently, peri-domestic populations of the Amazonian lineage were found in the region of the outbreak [100]. It is quite remarkable that a species that in its sylvatic habitat is a canopy dweller adapts to anthropogenic biomes. This probably occurred because the females found suitable oviposition sites that were rich in organic material in these biomes, resulting in the production of adult flies that subsequently increased the chances of their feeding on humans. The migration from sylvatic to anthropogenic biomes is well documented for *Lutzomyia* species such as *Lu. longipalpis*.

Determining the role of a sand fly in ACL and AVL epidemiology is complex. Over the years, various criteria have been used. Evidence of infection is primordial together with being attracted to humans. A recent publication evaluated the vectorial status of sand flies found infected in Colombia [96]. The authors created five vectorial levels that graduated from 1, based on just detecting parasite DNA, to 5 which was a proven vector. The vectorial statuses of the flies with infections reviewed in this paper fall within these five categories. However, the problem is that there are records in different countries where a species may be a vector in one but not in the other. Because of this difficulty, irrespective of the country, certain species have been highlighted as being proven vectors in Table 1. The vectorial situation of a sand fly species is further complicated when more than one species has been found to be infected with the same parasite in the same biome.

A study of Ecuadorian [41] sand flies using human landing collections showed marked differences in the dominance of different species collected from humans. In a coastal region, *Ps. maripaensis* represented 0.1% of the total, yet in a mountainous region, it was 9.7%. A more marked example was *Lu. ayacuchensis* that was 47.5% of the catch in the Andean region, and merely 1.4% in the Amazon region. At higher altitudes, *Lu. ayacuchensis* is an anthropportunist species, but if studies had been limited to the Amazonian lowlands, it could be categorized as an anthroaccidentalists due to the low numbers captured from humans. This indicates that relative attraction of phlebotomine species to humans can only be accurately assessed when their population dynamics are known. To do this, population densities must be evaluated simultaneously using different methods in different biomes.

Under the present-day ethical environment, the definitive solution of knowing whether a species feeds on humans is blood meal analysis using molecular methods. Beyond this, there is circumstantial evidence when the same parasite is found in both a sand fly and human in the same biome, but it is not definitive proof of it feeding on humans as other species could transmit the same parasite.

## Conclusion

Sand fly generalist or specialist status is related to the parasite species found in them and the hosts that they feed on. Of the 75 generalist species, 52 were considered anthropportunists, 5 anthroaccidentalists, and 18 zoophilic. The anthropportunists included important ACL and AVL vectors. Zoophilic species may participate in the maintenance of the leishmanial enzootic cycle. The *Leishmania* species associated with the 12 anthroaccidentalist species of the genera *Bichromomyia*, *Psathyromyia*, and *Trichophoromyi*a are less common in humans, but under specific environmental circumstances may become ACL vectors. The majority of records are based on molecular parasite identifications, so it is impossible to say whether they represent transmissible or dead-end infections. Importantly, finding a parasite in a sand fly solely indicates a potential vectorial role that needs to be investigated in greater detail.

As more precise information on the biology of sand fly species becomes available, it will be possible to fine tune these categories. Finding the same parasite in sand flies belonging to different categories poses many questions. Combinations of 157 individual *Leishmania* infections were recorded in 91 sand fly species (Table 1). These varied from infections of one *Leishmania* species to seven in a single sand fly species. There are records of *L.* (*V*.) *braziliensis* in 43 different sand fly species, of which 29 are anthropportunists, one is an anthroaccidentalist, and 12 are zoophilic species. Concerning the epidemiological significance of this, one possibility is that it generates the extensive genetic diversity of this species [51] and that the relative epidemiological importance of a particular sand fly species is associated with a specific biome. Also, mere records of parasites in a species do not indicate its vectorial or epidemiological importance, and zoophilic species of interest may be playing a part in the maintenance of the enzootic cycle. Infections of *L.* (*V*.) *naiffi* continue to be found [46] and it has now been recorded in 18 species belonging to the three different categories. Anecdotally, *Ps. ayrozai* has sometimes been selected as the main vector, as it was detected feeding on armadillos, but presumably the other infections were acquired feeding on armadillos or perhaps other animals. Similarly for *L.* (*V*.) *braziliensis*, it is likely that the extensive genetic diversity of the armadillo *Leishmania* is linked to its presence in many vectors. Such questions are associated with the species in which *L.* (*V*.) *guyanensis*, *L.* (*V*.) *panamensis*, and *L.* (*L*.) *infantum* have been found.

The problem with the terms “anthropophily” and “anthropophilic” is that they are overgeneralizations that unfortunately promote misleading simplifications of sand fly behavior. In relation to feeding, they falsely suggest a deliberate preference for humans when sand flies are opportunistic feeders, biting humans only when other hosts are unavailable. Such anthropocentric biases obscure the true ecological dynamics of vector-host interactions, ultimately weakening our understanding of disease transmission. The use of the generalist and specialist concept, and the introduction of the terms anthropportunist and anthroaccidentalist, are aimed at diminishing these distortions, fostering a more precise, evidence-based approach to evaluating sand fly behavior and its role in the intricate epidemiology web of leishmaniasis in the Americas.

## References

[1] Alexander B, Ferro C, Young DG, Morales A, Tesh RB. 1992. Ecology of phlebotomine sand flies (Diptera: Psychodidae) in a focus of *Leishmania* (*Viannia*) *braziliensis* in northeastern Colombia. Memórias do Instituto Oswaldo Cruz, 87(3), 387–395. 1343648 10.1590/s0074-02761992000300009

[2] Aragão HB. 1922. Transmissão da leishmaniose tegumentar no Brasil pelo *Phlebotomus intermedius*. Brasil Médico, 36, 129–130.

[3] Araujo-Pereira T, Pita-Pereira D, Boite MC, Melo M, Costa-Rego TA, Fuzari AA, Brazil RP, Britto C. 2017. First description of *Leishmania (Viannia)* infection in *Evandromyia saulensis*, *Pressatia* sp. and *Trichophoromyia auraensis* (Psychodidae: Phlebotominae) in a transmission area of cutaneous leishmaniasis in Acre state, Amazon Basin, Brazil. Memórias do Instituto Oswaldo Cruz, 112(1), 75–78. 28076470 10.1590/0074-02760160283 PMC5225531

[4] Arias JR, Miles MA, Naiff RD, Povoa MM, de Freitas RA, Biancardi CB, Castellon EG. 1985. Flagellate infections of Brazilian sand flies (Diptera: Psychodidae): isolation *in vitro* and biochemical identification of *Endotrypanum* and *Leishmania*. American Journal of Tropical Medicine and Hygiene, 34(6), 1098–1108. 3938924 10.4269/ajtmh.1985.34.1098

[5] Arrivillaga-Henriquez J, Enriquez S, Romero V, Echeverria G, Perez-Barrera J, Poveda A, Navarro JC, Warburg A, Benitez W. 2017. Eco-epidemiological aspects, natural detection and molecular identification of *Leishmania* spp. in *Lutzomyia reburr*a, *Lutzomyia barrettoi majuscul*a and *Lutzomyia trapidoi*. Biomedica, 37(2), 83–97. 29161481 10.7705/biomedica.v37i0.3536

[6] Azevedo AC, Rangel EF, Costa EM, David J, Vasconcelos AW, Lopes UG. 1990. Natural infection of *Lutzomyia* (*Nyssomyia*) *whitmani* (Antunes and Coutinho, 1939) by *Leishmania* of the *braziliensis* complex in Baturit‚ Ceará State, Northeast Brazil. Memórias do Instituto Oswaldo Cruz, 85, 251. 2087162 10.1590/s0074-02761990000200021

[7] Azevedo AC, Rangel EF, Queiroz RG. 1990. *Lutzomyia migonei* (Franca, 1920) naturally infected with peripylarian flagellates in Baturité, a focus of cutaneous leishmaniasis in Ceará state, Brazil. Memórias do Instituto Oswaldo Cruz, 85(4), 479. 2152202 10.1590/s0074-02761990000400016

[8] Azpurua J, De La Cruz D, Valderama A, Windsor D. 2010. *Lutzomyia* sand fly diversity and rates of infection by *Wolbachia* and an exotic *Leishmania* species on Barro Colorado Island, Panama. PLoS Neglected Tropical Diseases, 4(3), e627. 20231892 10.1371/journal.pntd.0000627 PMC2834748

[9] Bastrenta B, Buitrago R, Vargas F, Le Pont F, Torrez M, Flores M, Mita N, Breniere SF. 2002. First evidence of transmission of *Leishmania* (*Viannia*) *lainsoni* in a Sub Andean region of Bolivia. Acta Tropica, 83(3), 249–253. 12204398 10.1016/s0001-706x(02)00129-8

[10] Baum M, Ribeiro MC, Lorosa ES, Damasio GA, Castro EA. 2013. Eclectic feeding behavior of *Lutzomyia* (*Nyssomyia*) *intermedia* (Diptera, Psychodidae, Phlebotominae) in the transmission area of American cutaneous leishmaniasis, state of Parana, Brazil. Revista da Sociedade Brasileira de Medicina Tropical, 46(5), 560–565. 24270247 10.1590/0037-8682-0157-2013

[11] Biagi FF, De Biagi AM, Beltran HF. 1965. *Phlebotomus flaviscutellatus,* transmisor natural de *Leishmania mexicana*. Prensa Médica de Mexicana, 30, 267–272. 5598515

[12] Braga RR, Lainson R, Ishikawa EA, Shaw JJ. 2003. *Leishmania* (*Viannia*) *utingensis* n. sp., a parasite from the sandfly *Lutzomyia* (*Viannamyia*) *tuberculata* in Amazonian Brazil. Parasite, 10(2), 111–118. 12847917 10.1051/parasite/2003102111

[13] Braga RR, Lainson R, Shaw JJ, Ryan L, Silveira FT. 1986. Leishmaniasis in Brazil. XXII: Characterization of *Leishmania* from man, dogs and the sandfly *Lutzomyia longipalpis* (Lutz & Neiva, 1912) isolated during an outbreak of visceral leishmaniasis in Santarem, Para State. Transactions of the Royal Society of Tropical Medicine and Hygiene, 80(1), 143–145. 3726975 10.1016/0035-9203(86)90214-2

[14] Bueno AC, Machado VE, da Rocha Silva FB, Boni FI, Cury BSF, Gremiao MPD, Pinto MC. 2023. Semiochemical delivery systems based on natural polymers to attract sand flies (Diptera: Psychodidae). Parasites & Vectors, 16(1), 303. 37644584 10.1186/s13071-023-05931-w PMC10464299

[15] Caceres AG, Villaseca P, Dujardin JC, Banuls AL, Inga R, Lopez M, Arana M, Le Ray D, Arevalo J. 2004. Epidemiology of Andean cutaneous leishmaniasis: incrimination of *Lutzomyia ayacuchensis* (Diptera: Psychodidae) as a vector of *Leishmania* in geographically isolated, upland valleys of Peru. American Journal of Tropical Medicine and Hygiene, 70(6), 607–612. 15211000

[16] Campbell-Lendrum DH, Brandao-Filho SP, Ready PD, Davies CR. 1999. Host and/or site loyalty of *Lutzomyia whitman*i (Diptera: Psychodidae) in Brazil. Medical and Veterinary Entomology, 13(2), 209–211. 10484168 10.1046/j.1365-2915.1999.00169.x

[17] Campbell-Lendrum DH, Pinto MC, Brandao-Filho SP, de Souza AA, Ready PD, Davies CR. 1999. Experimental comparison of anthropophily between geographically dispersed populations of *Lutzomyia whitmani* (Diptera: Psychodidae). Medical and Veterinary Entomology, 13(3), 299–309. 10514057 10.1046/j.1365-2915.1999.00174.x

[18] Caneda-Guzman IC, Oca-Aguilar ACM, Miranda-Caballero CI, Grostieta E, Correa-Morales F, Romero-Perez R, Romero-Contreras FE, Rodriguez-Atanacio JA, Ruiz-Tovar K, Huerta H, Mis-Avila PC, Quintanilla-Cedillo MR, Lammoglia-Villagomez MA, Blum-Dominguez S, Tamay-Segovia P, Rojas-Ronquillo R, Sanchez-Montes S, Becker I. 2023.Entomological survey and *Leishmania* (*Leishmania*) *mexicana* prevalence in sand fly species during an outbreak of cutaneous leishmaniasis in Quintana Roo State, Mexico. Tropical Medicine and Infectious Disease, 8(10), 465. 37888593 10.3390/tropicalmed8100465 PMC10610947

[19] Carvalho GM, Andrade Filho JD, Falcao AL, Rocha Lima AC, Gontijo CM. 2008. Naturally infected *Lutzomyia* sand flies in a *Leishmania*-endemic area of Brazil. Vector-Borne and Zoonotic Diseases, 8(3), 407–414. 18429695 10.1089/vbz.2007.0180

[20] Carvalho GM, Brazil RP, Rego FD, Ramos MC, Zenobio AP, Andrade Filho JD. 2017. Molecular detection of *Leishmania* DNA in wild-caught phlebotomine sand flies (Diptera: Psychodidae) from a cave in the State of Minas Gerais, Brazil. Journal of Medical Entomology, 54(1), 196–203. 28082647 10.1093/jme/tjw137

[21] Cook LM, Saccheri IJ. 2013. The peppered moth and industrial melanism: evolution of a natural selection case study. Heredity 110(3), 207–212. 23211788 10.1038/hdy.2012.92 PMC3668657

[22] Costa GDS, Junior AMP, Castro TS, de Paulo PFM, Ferreira GEM, Medeiros JF. 2021. Sand fly fauna and molecular detection of *Leishmania* species and blood meal sources in different rural environments in western Amazon. Acta Tropica, 224, 106150. 34562421 10.1016/j.actatropica.2021.106150

[23] Davies CR, Fernandez M, Paz L, Roncal N, Llanos-Cuentas A. 1993. *Lutzomyia verrucarum* can transmit *Leishmania peruviana*, the aetiological agent of Andean cutaneous leishmaniasis. Transactions of the Royal Society of Tropical Medicine and Hygiene, 87(5), 603–606. 8266422 10.1016/0035-9203(93)90103-w

[24] de Avila MM, Brilhante AF, de Souza CF, Bevilacqua PD, Galati EAB, Brazil RP. 2018. Ecology, feeding and natural infection by *Leishmania* spp. of phlebotomine sand flies in an area of high incidence of American tegumentary leishmaniasis in the municipality of Rio Branco, Acre, Brazil. Parasites & Vectors, 11(1), 64. 29373995 10.1186/s13071-018-2641-y PMC5787322

[25] de Pita-Pereira D, Cardoso MA, Alves CR, Brazil RP, Britto C. 2008. Detection of natural infection in *Lutzomyia cruzi* and *Lutzomyia forattini*i (Diptera: Psychodidae: Phlebotominae) by *Leishmania infantum chagasi* in an endemic area of visceral leishmaniasis in Brazil using a PCR multiplex assay. Acta Tropica, 107(1), 66–69. 18502392 10.1016/j.actatropica.2008.04.015

[26] de Souza A, Ishikawa E, Braga R, Silveira F, Lainson R, Shaw J. 1996. *Psychodopygus complexus*, a new vector of *Leishmania braziliensis* to humans in Pará State, Brazil. Transactions of the Royal Society of Tropical Medicine and Hygiene, 90(2), 112–113. 8761563 10.1016/s0035-9203(96)90103-0

[27] de Souza AA, Dos Santos TV, Jennings YL, Ishikawa EA, Barata ID, Silva MD, Lima JA, Shaw J, Lainson R, Silveira FT. 2016. Natural *Leishmania* (*Viannia)* spp. infections in phlebotomine sand flies (Diptera: Psychodidae) from the Brazilian Amazon region reveal new putative transmission cycles of American cutaneous leishmaniasis. Parasite, 23, 22. 27235194 10.1051/parasite/2016022 PMC4884270

[28] de Souza AAA, da Rocha Barata I, das Gracas Soares Silva M, Lima JAN, Jennings YLL, Ishikawa EAY, Prevot G, Ginouves M, Silveira FT, Shaw J, dos Santos TV. 2017. Natural *Leishmania* (*Viannia*) infections of phlebotomines (Diptera: Psychodidae) indicate classical and alternative transmission cycles of American cutaneous leishmaniasis in the Guiana Shield, Brazil. Parasite, 24, 13. 28508745 10.1051/parasite/2017016 PMC5432964

[29] Donalisio MR, Paiz LM, da Silva VG, Richini-Pereira VB, von Zuben APB, Castagna CL, Motoie G, Hiramoto RM, Tolezano JE. 2017. Visceral leishmaniasis in an environmentally protected area in southeastern Brazil: Epidemiological and laboratory cross-sectional investigation of phlebotomine fauna, wild hosts and canine cases. PLoS Neglected Tropical Diseases, 11(7), e0005666. 28704391 10.1371/journal.pntd.0005666 PMC5509102

[30] Dvorak V, Shaw J, Volf P. 2018. Parasite biology: the vectors, in: The Leishmaniases: old neglected tropical diseases, Bruschi F, Gradoni L, Editors. Springer International Publishing: Basel, p. 31–77.

[31] Fairchild GB, Hertig M. 1948. Notes on the *Phlebotomus* of Panama (Diptera, Psychodidae) III. *P. cruciatus* Coq., *trinidadensis* Newst. and *gomezi* Nitz. Annals of the Entomological Society of America, 41(2), 247–257.

[32] Fairchild GB, Hertig M. 1952. Notes on the *Phlebotomus* of Panama IX. Descriptions of seven new species. Annals of the Entomological Society of America, 45, 505–528.

[33] Feliciangeli MD, Rabinovich J. 1998. Abundance of *Lutzomyia ovallesi* but not *Lu. gomezi* (Diptera: Psychodidae) correlated with cutaneous leishmaniasis incidence in north-central Venezuela. Medical and Veterinary Entomology, 12(2), 121–131. 9622364 10.1046/j.1365-2915.1998.00072.x

[34] Ferreira RC, de Souza AA, Freitas RA, Campaner M, Takata CS, Barrett TV, Shaw JJ, Teixeira MM. 2008. A phylogenetic lineage of closely related trypanosomes (Trypanosomatidae, Kinetoplastida) of anurans and sand flies (Psychodidae, Diptera) sharing the same ecotopes in Brazilian Amazonia. Journal of Eukaryotic Microbiology, 55(5), 427–435. 19017063 10.1111/j.1550-7408.2008.00342.x

[35] Ferro C, Marin D, Gongora R, Carrasquilla MC, Trujillo JE, Rueda NK, Marin J, Valderrama-Ardila C, Alexander N, Perez M, Munstermann LE, Ocampo CB. 2011. Phlebotomine vector ecology in the domestic transmission of American cutaneous leishmaniasis in Chaparral, Colombia. American Journal of Tropical Medicine and Hygiene, 85(5), 847–856. 22049038 10.4269/ajtmh.2011.10-0560 PMC3205630

[36] Galati EAB. 2018. Phlebotominae (Diptera, Psychodidae): classification, morphology and terminology of adults and identification, in: Brazilian Sand Flies, Rangel EF, Shaw JJ, Editors. Springer: Basel, p. 9–212.

[37] Galvis-Ovallos F, da Silva MD, Bispo GB, de Oliveira AG, Neto JR, Malafronte RD, Galati EA. 2017. Canine visceral leishmaniasis in the metropolitan area of Sao Paulo: *Pintomyia fischeri* as potential vector of *Leishmania infantum*. Parasite, 24, 2. 28134092 10.1051/parasite/2017002 PMC5780806

[38] Garcia AL, Tellez T, Parrado R, Rojas E, Bermudez H, Dujardin JC. 2007. Epidemiological monitoring of American tegumentary leishmaniasis: molecular characterization of a peridomestic transmission cycle in the Amazonian lowlands of Bolivia. Transactions of the Royal Society of Tropical Medicine and Hygiene, 101, 1208–1213. 17945322 10.1016/j.trstmh.2007.09.002

[39] Gil LH, Basano SA, Souza AA, Silva MG, Barata I, Ishikawa EA, Camargo LM, Shaw JJ. 2003. Recent observations on the sand fly (Diptera: Psychodidae) fauna of the State of Rondonia, Western Amazonia, Brazil: the importance of *Psychdopygus davisi* as a vector of zoonotic cutaneous leishmaniasis. Memórias do Instituto Oswaldo Cruz, 98(6), 751–755. 14595450 10.1590/s0074-02762003000600007

[40] Gomes AC. 1994. Sand fly vectorial ecology in the State of Sao Paulo. Memórias do Instituto Oswaldo Cruz, 89(3), 457–460. 7476232 10.1590/s0074-02761994000300030

[41] Gomez EA, Kato H, Hashiguchi Y. 2014. Man-biting sand fly species and natural infection with the *Leishmania* promastigote in leishmaniasis-endemic areas of Ecuador. Acta Tropica, 140, 41–49. 25063388 10.1016/j.actatropica.2014.07.003

[42] Goncalves LP, Santos TVD, Campos MB, Lima L, Ishikawa EAY, Silveira FT, Ramos PKS. 2020. Further insights into the eco-epidemiology of American cutaneous leishmaniasis in the Belem metropolitan region, Para State, Brazil. Revista da Sociedade Brasileira de Medicina Tropical, 53, e20200255. 33331607 10.1590/0037-8682-0255-2020 PMC7747830

[43] Gonzalez C, Leon C, Paz A, Lopez M, Molina G, Toro D, Ortiz M, Cordovez JM, Atencia MC, Aguilera G, Tovar C. 2018. Diversity patterns, *Leishmania* DNA detection, and bloodmeal identification of Phlebotominae sand flies in villages in northern Colombia. PLoS One, 13(1), e0190686. 29320544 10.1371/journal.pone.0190686 PMC5761875

[44] Gonzalez C, Rebollar-Tellez EA, Ibanez-Bernal S, Becker-Fauser I, Martinez-Meyer E, Peterson AT, Sanchez-Cordero V. 2011. Current knowledge of *Leishmania* vectors in Mexico: how geographic distributions of species relate to transmission areas. American Journal of Tropical Medicine and Hygiene, 85(5), 839–846. 22049037 10.4269/ajtmh.2011.10-0452 PMC3205629

[45] Grimaldi G Jr, Momen H, Naiff RD, McMahon-Pratt D, Barrett TV. 1991. Characterization and classification of leishmanial parasites from humans, wild mammals, and sand flies in the Amazon region of Brazil. American Journal of Tropical Medicine and Hygiene, 44(6), 645–661. 1858968 10.4269/ajtmh.1991.44.645

[46] Guimaraes RCS, Marialva EF, Feijo JA, Pereira-Silva JW, Martins-Campos KM, Gontijo CMF, Pereira AAS, Rios-Velasquez CM, Pessoa FAC. 2022. Trypanosomatids in phlebotomine sand flies (Diptera: Phlebotominae) from anthropic and sinantropic landscapes in a rural settlement in the Brazilian Amazon. Journal of Medical Entomology, 59(2), 681–692. 35022773 10.1093/jme/tjab208

[47] Guimaraes VC, Costa PL, Silva FJ, Melo FL, Dantas-Torres F, Rodrigues EH, Brandao Filho SP. 2014. Molecular detection of *Leishmania* in phlebotomine sand flies in a cutaneous and visceral leishmaniasis endemic area in northeastern Brazil. Revista do Instituto de Medicina Tropical de São Paulo, 56(4), 357–360. 25076439 10.1590/S0036-46652014000400015 PMC4131824

[48] Hashiguchi Y, Gomez EA, de Coronel VV, Mimori T, Kawabata M, Furuya M, Nonaka S, Takaoka H, Alexander JB, Quizhpe AM, Grimaldi G, Kreutzer RD, Tesh RB. 1991. Andean leishmaniasis in Ecuador caused by infection with *Leishmania mexicana* and *L. major*-like parasites. American Journal of Tropical Medicine and Hygiene, 44(2), 205–217. 1672799 10.4269/ajtmh.1991.44.205

[49] Hashiguchi Y, Gomez LEA, Cáceres AG, Velez LN, Villegas NV, Hashiguchi K, Mimori T, Uezato H, Kato H. 2018. Andean cutaneous leishmaniasis (Andean-CL, uta) in Peru and Ecuador: the vector *Lutzomyia* sand flies and reservoir mammals. Acta Tropica, 178, 264–275. 29224978 10.1016/j.actatropica.2017.12.008

[50] Hashiguchi Y, Hashiguchi K, Zambrano FC, Parraga FD, Martillo VP, Torres EX, Velez LN, Villegas NV, Gomez EA, Kato H. 2020. Natural *Leishmania* (*Leishmania*) *mexicana* infection and biting activity of anthropophilic sand fly *Lutzomyia ayacuchensis* in the Ecuadorian Andes. Acta Tropica, 203, 105321. 31877283 10.1016/j.actatropica.2019.105321

[51] Heeren S, Sanders M, Shaw JJ, Pinto Brandao-Filho S, Cortes Boite M, Motta Cantanhede L, Chourabi K, Maes I, Llanos-Cuentas A, Arevalo J, Marco JD, Lemey P, Cotton JA, Dujardin JC, Cupolillo E, Van den Broeck F. 2024. Evolutionary genomics of *Leishmania braziliensis* across the neotropical realm. Communications Biology, 7(1), 1587. 39609617 10.1038/s42003-024-07278-z PMC11605123

[52] Hoyos J, Gonzalez R, Cuellar ME, Leon C. 2020. Ecology of sand flies (Psychodidae: Phlebotominae) and natural infection of *Pintomyia townsendi* with *Leishmania amazonensis* in a cutaneous leishmaniasis focus in Colombia. Journal of Medical Entomology, 57(5), 1653–1658. 32222761 10.1093/jme/tjaa056

[53] Jorquera A, Gonzalez R, Marchan-Marcano E, Oviedo M, Matos M. 2005. Multiplex-PCR for detection of natural *Leishmania* infection in *Lutzomyia* spp. captured in an endemic region for cutaneous leishmaniasis in state of Sucre, Venezuela. Memórias do Instituto Oswaldo Cruz, 100(1), 45–48. 15867962 10.1590/s0074-02762005000100008

[54] Karakus M, Gocmen B, Ozbel Y. 2017. Insecticide susceptibility status of wild-caught sand fly populations collected from two leishmaniasis endemic areas in Western Turkey. Journal of Arthropod-Borne Diseases, 11(1), 86–94. 29026855 PMC5629309

[55] Kato H, Caceres AG, Hashiguchi Y. 2016. First evidence of a hybrid of *Leishmania* (*Viannia*) *braziliensis*/*L*. (*V*.) *peruviana* DNA detected from the phlebotomine sand fly *Lutzomyia tejadai* in Peru. PLoS Neglected Tropical Diseases, 10(1), e0004336. 26735142 10.1371/journal.pntd.0004336 PMC4703407

[56] Kato H, Calvopina M, Criollo H, Hashiguchi Y. 2013. First human cases of *Leishmani*a (*Viannia*) *naiffi* infection in Ecuador and identification of its suspected vector species. Acta Tropica, 128(3), 710–713. 24044975 10.1016/j.actatropica.2013.09.001

[57] Kato H, Gomez EA, Yamamoto Y, Calvopina M, Guevara AG, Marco JD, Barroso PA, Iwata H, Hashiguchi Y. 2008. Natural infection of *Lutzomyia tortura* with *Leishmania* (*Viannia*) *naiffi* in an Amazonian area of Ecuador. American Journal of Tropical Medicine and Hygiene, 79(3), 438–440. 18784239

[58] Kato H, Uezato H, Katakura K, Calvopina M, Marco JD, Barroso PA, Gomez EA, Mimori T, Korenaga M, Iwata H, Nonaka S, Hashiguchi Y. 2005. Detection and identification of *Leishmani*a species within naturally infected sand flies in the andean areas of Ecuador by a polymerase chain reaction. American Journal of Tropical Medicine and Hygiene, 72(1), 87–93. 15728872

[59] Kreutzer RD, Corredor A, Grimaldi G Jr, Grogl M, Rowton ED, Young DG, Morales A, McMahon-Pratt D, Guzman H, Tesh RB. 1991. Characterization of *Leishmania colombiensis* sp. n. (Kinetoplastida: Trypanosomatidae), a new parasite infecting humans, animals, and phlebotomine sand flies in Colombia and Panama. American Journal of Tropical Medicine and Hygiene, 44(6), 662–675. 1677544 10.4269/ajtmh.1991.44.662

[60] Lainson R. 1988. Ecological interactions in the transmission of the leishmaniases. Philosophical Transactions of the Royal Society B, 321, 389–404. 10.1098/rstb.1988.0099 2907150

[61] Lainson R, Braga RR, de Souza AA, Póvoa MM, Ishikawa EA, Silveira FT. 1989. *Leishmania* (*Viannia*) *shawi* n. sp., a parasite of monkeys, sloths and procyonids in Amazonian Brazil. Annales de Parasitologie Humaine et Comparée, 64, 200–207. 2504099 10.1051/parasite/1989643200

[62] Lainson R, Shaw JJ. 1968. Leishmaniasis in Brazil: I. Observations on enzootic rodent leishmaniasis–incrimination of *Lutzomyia flaviscutellata* (Mangabeira) as the vector in the Lower Amazonian Basin. Transactions of the Royal Society of Tropical Medicine and Hygiene, 62(3), 385–395. 5659232 10.1016/0035-9203(68)90090-4

[63] Lainson R, Shaw JJ, Souza AA, Silveira FT, Falqueto A. 1992. Further observations on *Lutzomyi*a *ubiquitalis* (Psychodidae: Phlebotominae), the sandfly vector of *Leishmania* (*Viannia*) *lainsoni*. Memórias do Instituto Oswaldo Cruz, 87(3), 437–439. 1343653 10.1590/s0074-02761992000300016

[64] Lainson R, Shaw JJ, Ward RD, Fraiha H. 1973. Leishmaniasis in Brazil. IX. Considerations on the *Leishmania braziliensis* complex. Importance of sandflies of the genus *Psychodopygus* (Mangabeira) in the transmission of *L. braziliensis braziliensis* in north Brazil. Transactions of the Royal Society of Tropical Medicine and Hygiene, 67(2), 184–196. 4784055 10.1016/0035-9203(73)90143-0

[65] Lainson R, Ward RD, Shaw JJ. 1977. Experimental transmission of *Leishmania chagasi*, causative agent of neotropical visceral leishmaniasis, by the sandfly *Lutzomyia longipalpis*. Nature, 266(5603), 628–630. 859627 10.1038/266628a0

[66] Lara-Silva Fde O, Michalsky EM, Fortes-Dias EM, Fiuza Vde O, Pessanha JE, Regina-Silva S, de Avelar DM, Silva MA, Lima AC, da Costa AJ, Machado-Coelho GL, Dias ES. 2015. Epidemiological aspects of vector, parasite, and domestic reservoir in areas of recent transmission and no reported human cases of visceral leishmaniasis in Brazil. Acta Tropica, 148, 128–136. 25882769 10.1016/j.actatropica.2015.04.002

[67] Le Pont F, Desjeux P. 1986. Leishmaniasis in Bolivia. II. The involvement of *Psychodopygus yucumensis* and *Psychodopygus llanosmartinsi* in the selvatic transmission cycle of *Leishmania braziliensis braziliensis* in a lowland subandean region. Memórias do Instituto Oswaldo Cruz, 81(3), 311–318. 3574129 10.1590/s0074-02761986000300007

[68] Le Pont F, Leon R, Guerrini F, Gantier JC, Mouchet J, Echeverria R, Guderian RH. 1994. Leishmaniose en Équateur. 3. *Lutzomyia trapidoi*, vecteur de *Leishmania panamensis*. Annales de la Société Belge de Médecine Tropicale, 74(1), 23–28. 8024346

[69] Leao PO, Pereira Junior AM, de Paulo PFM, Carvalho LPC, Souza ABN, da Silva MS, Castro TS, Freitas MTS, Rodrigues MMS, Ferreira GEM, Medeiros JF. 2020. Vertical stratification of sand fly diversity in relation to natural infections of *Leishmania* sp. and blood-meal sources in Jamari National Forest, Rondonia State, Brazil. Parasites & Vectors, 13(1), 422. 32807221 10.1186/s13071-020-04295-9 PMC7433131

[70] Liu N. 2015. Insecticide resistance in mosquitoes: impact, mechanisms, and research directions. Annual Review of Entomology, 60, 537–559. 10.1146/annurev-ento-010814-020828 25564745

[71] Lopez M, Erazo D, Hoyos J, Leon C, Fuya P, Lugo L, Cordovez JM, Gonzalez C. 2021. Measuring spatial co-occurrences of species potentially involved in *Leishmania* transmission cycles through a predictive and fieldwork approach. Scientific Reports, 11(1), 6789. 33762622 10.1038/s41598-021-85763-9 PMC7990927

[72] Marcondes CB, Bittencourt IA, Stoco PH, Eger I, Grisard EC, Steindel M. 2009. Natural infection of *Nyssomyia neivai* (Pinto, 1926) (Diptera: Psychodidae, Phlebotominae) by *Leishmania* (*Viannia*) spp. in Brazil. Transactions of the Royal Society of Tropical Medicine and Hygiene, 103(11), 1093–1097. 19178921 10.1016/j.trstmh.2008.12.006

[73] Marcondes CB, Day JC, Ready PD. 1997. Introgression between *Lutzomyia intermedi*a and both *Lu. neivai* and *Lu. whitman*i, and their roles as vectors of *Leishmania braziliensi*s. Transactions of the Royal Society of Tropical Medicine and Hygiene, 91(6), 725–726. 9509190 10.1016/s0035-9203(97)90540-x

[74] Marinho-Junior JF, Monteiro J, Sales de Carvalho AW, de Carvalho FG, de Paiva Cavalcanti M, Shaw J, Courtenay O, Brandao-Filho SP. 2023. High levels of infectiousness of asymptomatic *Leishmania* (*Viannia*) *braziliensis* infections in wild rodents highlights their importance in the epidemiology of American tegumentary leishmaniasis in Brazil. PLoS Neglected Tropical Diseases, 17(1), e0010996. 36716345 10.1371/journal.pntd.0010996 PMC9910795

[75] Martinez E, Le Pont F, Torrez M, Telleria J, Vargas F, Dujardin JC, Dujardin JP. 1999. *Lutzomyia nuneztovari anglesi* (Le Pont & Desjeux, 1984) as a vector of *Leishmania amazonensis* in a sub-Andean leishmaniasis focus of Bolivia. American Journal of Tropical Medicine and Hygiene, 61(5), 846–849. 10586923 10.4269/ajtmh.1999.61.846

[76] Martinez E, Le Pont F, Torrez M, Telleria J, Vargas F, Munoz M, De Doncker S, Dujardin JC, Dujardin JP. 1998. A new focus of cutaneous leishmaniasis due to *Leishmania amazonensis* in a sub Andean region of Bolivia. Acta Tropica, 71(2), 97–106. 9821459 10.1016/s0001-706x(98)00049-7

[77] McConnell E. 1963. Leptomonads of wild-caught Panamanian *Phlebotomus*: Culture and animal inoculation. Experimental Parasitology, 14, 123–128. 14053097 10.1016/0014-4894(63)90016-x

[78] McHugh CP, Grogl M, Kreutzer RD. 1993. Isolation of *Leishmania mexicana* (Kinetoplastida: Trypanosomatidae) from *Lutzomyia anthophora* (Diptera: Psychodidae) collected in Texas. Journal of Medical Entomology, 30(3), 631–633. 8510126 10.1093/jmedent/30.3.631

[79] Mejia A, Matamoros G, Fontecha G, Sosa-Ochoa W. 2018. Bionomic aspects of *Lutzomyia evansi* and *Lutzomyia longipalpis,* proven vectors of *Leishmania infantum* in an endemic area of non-ulcerative cutaneous leishmaniasis in Honduras. Parasites & Vectors, 11(1), 15. 29304878 10.1186/s13071-017-2605-7 PMC5756426

[80] Miranda A, Carrasco R, Paz H, Pascale JM, Samudio F, Saldana A, Santamaria G, Mendoza Y, Calzada JE. 2009. Molecular epidemiology of American tegumentary leishmaniasis in Panama. American Journal of Tropical Medicine and Hygiene, 81(4), 565–571. 19815867 10.4269/ajtmh.2009.08-0265

[81] Montes de Oca-Aguilar AC, Fernandez-Figueroa EA, Lopez-Avila KB, Pavon-Mendez MI, Sosa-Bibiano EI, Rebollar-Tellez EA, Palacio-Vargas JA, Garcia-Lopez B, Rangel-Escareno C, Loria-Cervera EN. 2024. Abundance and *Leishmania* infection patterns of the sand fly *Psathyromyia cratife*r in Southern Mexico. PLoS Neglected Tropical Diseases, 18(9), e0012426. 39255321 10.1371/journal.pntd.0012426 PMC11414901

[82] Moo-Llanes DA, Arque-Chunga W, Carmona-Castro O, Yanez-Arenas C, Yanez-Trujillano HH, Cheverria-Pacheco L, Baak-Baak CM, Caceres AG. 2017. Shifts in the ecological niche of *Lutzomyia peruensis* under climate change scenarios in Peru. Medical and Veterinary Entomology, 31(2), 123–131. 28150865 10.1111/mve.12219

[83] Moreno M, Ferro C, Rosales-Chilama M, Rubiano L, Delgado M, Cossio A, Gomez MA, Ocampo C, Saravia NG. 2015. First report of *Warileya rotundipennis* (Psychodidae: Phlebotominae) naturally infected with *Leishmania* (*Viannia*) in a focus of cutaneous leishmaniasis in Colombia. Acta Tropica, 148, 191–196. 25917717 10.1016/j.actatropica.2015.04.017 PMC4654406

[84] Morrison AC, Ferro C, Tesh RB. 1993. Host preferences of the sand fly *Lutzomyia longipalpis* at an endemic focus of American visceral leishmaniasis in Colombia. American Journal of Tropical Medicine and Hygiene, 49(1), 68–75. 8352394 10.4269/ajtmh.1993.49.68

[85] Moya SL, Giuliani MG, Manteca Acosta M, Salomon OD, Liotta DJ. 2015. First description of *Migonemyia migonei* (Franca) and *Nyssomyia whitmani* (Antunes & Coutinho) (Psychodidae: Phlebotominae) natural infected by *Leishmania infantum* in Argentina. Acta Tropica, 152, 181–184. 26409011 10.1016/j.actatropica.2015.09.015

[86] Nery-Guimaraes F. 1955. A study of a focus of mucocutaneous leishmaniasis in lowlands of the state of Rio de Janeiro. Memórias do Instituto Oswaldo Cruz, 53(1), 1–11. 13265230 10.1590/s0074-02761955000100001

[87] Paiva BR, Oliveira AG, Dorval ME, Galati EA, Malafronte RS. 2010. Species-specific identification of *Leishmania* in naturally infected sand flies captured in Mato Grosso do Sul State, Brazil. Acta Tropica, 115(1–2), 126–130. 20219438 10.1016/j.actatropica.2010.02.013

[88] Pech-May A, Escobedo-Ortegon FJ, Berzunza-Cruz M, Rebollar-Tellez EA. 2010. Incrimination of four sandfly species previously unrecognized as vectors of *Leishmania* parasites in Mexico. Medical and Veterinary Entomology, 24(2), 150–161. 20604861 10.1111/j.1365-2915.2010.00870.x

[89] Pech-May A, Peraza-Herrera G, Moo-Llanes DA, Escobedo-Ortegon J, Berzunza-Cruz M, Becker-Fauser I, Montes DEO-AAC, Rebollar-Tellez EA. 2016. Assessing the importance of four sandfly species (Diptera: Psychodidae) as vectors of *Leishmania mexicana* in Campeche, Mexico. Medical and Veterinary Entomology, 30(3), 310–320. 27040367 10.1111/mve.12169

[90] Penna HA. 1934. Leishmaniose visceral no Brasil. Brasil Médico, 48, 949–950.

[91] Perez JE, Villaseca P, Caceres A, Lopez M, Zolessi A, Campos M, Guerra H, Llanos-Cuentas A. 1991. *Leishmania* (*Viannia*) *peruvian*a isolated from the sandfly *Lutzomyia peruensis* (Diptera: Psychodidae) and a sentinel hamster in the Huayllacallan Valley, Ancash, Peru. Transactions of the Royal Society of Tropical Medicine and Hygiene, 85(1), 60. 2068762 10.1016/0035-9203(91)90158-u

[92] Perruolo G, Noris Rodriguez N, Feliciangeli MD. 2006. Isolation of *Leishmania* (*Viannia*) *braziliensis* from *Lutzomyia spinicrassa* (species group Verrucarum) Morales Osorno Mesa, Osorno and Hoyos 1969, in the Venezuelan Andean region. Parasite, 13(1), 17–22. 16605063 10.1051/parasite/2006131017

[93] Pessôa SB, Pestana BR. 1940. Infecção natural de “*Phlebotomus migonei*” por formas em leptomonas provavelmente da “*Leishmania brasiliensis*”. Acta medica (Rio de Janeiro), 5, 106–111.

[94] Pita-Pereira D, Souza GD, Zwetsch A, Alves CR, Britto C, Rangel EF. 2009. First report of *Lutzomyia* (*Nyssomyia*) *neivai* (Diptera: Psychodidae: Phlebotominae) naturally infected by *Leishmania* (*Viannia*) *braziliensis* in a periurban area of south Brazil using a multiplex polymerase chain reaction assay. American Journal of Tropical Medicine and Hygiene, 80(4), 593–595. 19346382

[95] Porter CH, Steurer FJ, Kreutzer RD. 1987. Isolation of *Leishmania mexicana mexicana* from *Lutzomyia ylephiletor* in Guatemala. Transactions of the Royal Society of Tropical Medicine and Hygiene, 81(6), 929–930. 3503412 10.1016/0035-9203(87)90355-5

[96] Posada-Lopez L, Galati EA, Shaw J, Galvis-Ovallos F. 2024. Incriminating leishmaniases vectors in Colombia: an overview and roadmap for future research. Acta Tropica, 260, 107409. 39317308 10.1016/j.actatropica.2024.107409

[97] Quinnell RJ, Dye C, Shaw JJ. 1992. Host preferences of the phlebotomine sandfly *Lutzomyia longipalpis* in Amazonian Brazil. Medical and Veterinary Entomology, 6(3), 195–200. 1421498 10.1111/j.1365-2915.1992.tb00606.x

[98] Quiroga C, Cevallos V, Morales D, Baldeón ME, Cárdenas P, Rojas-Silva P, Ponce P. 2017. Molecular identification of *Leishmania* spp. in sand flies (Diptera: Psychodidae, Phlebotominae) from Ecuador. Journal of Medical Entomology, 54(6), 1704–1711. 28981860 10.1093/jme/tjx122 PMC5850347

[99] Ready PD, Day JC, de Souza AA, Rangel EF, Davies CR.1997. Mitochondrial DNA characterization of populations of *Lutzomyia whitmani* (Diptera: Psychodidae) incriminated in the peri-domestic and silvatic transmission of *Leishmania* species in Brazil. Bulletin of Entomological Research, 87(2), 187–195.

[100] Ready PD, de Souza AA, Rebelo JM, Day JC, Silveira FT, Campbell-Lendrum D, Davies CR, Costa JM. 1998. Phylogenetic species and domesticity of *Lutzomyia whitmani* at the southeast boundary of Amazonian Brazil. Transactions of the Royal Society of Tropical Medicine and Hygiene, 92(2), 159–160. 9764319 10.1016/s0035-9203(98)90726-x

[101] Ready PD, Lainson R, Shaw JJ. 1984. *Leishmania (Viannia) braziliensis* does not develop in experientally infected *Bichromomyia flaviscutellata*. Unpublished manuscript.

[102] Ready PD, Lainson R, Shaw JJ, Souza AA. 1991. DNA probes for distinguishing *Psychodopygu*s *wellcomei* from *Psychodopygus complexus* (Diptera:Psychodidae). Memórias do Instituto Oswaldo Cruz, 86(1), 41–49. 1842400 10.1590/s0074-02761991000100008

[103] Ready PD, Lainson R, Shaw JJ, Ward RD. 1986. The ecology of *Lutzomyia umbratilis* Ward & Fraiha (Diptera Pyschodidae), the major vector to man of *Leishmania braziliensis guyanensis* Floch in northeast Amazonian Brazil. Bulletin of Entomological Research, 76, 21–40.

[104] Rebollar-Tellez EA, Reyes-Villanueva F, Fernandez-Salas I, Andrade-Narvaez FJ. 1996. Population dynamics and biting rhythm of the anthropophilic sandfly *Lutzomyia cruciata* (Diptera: Psychodidae) in Southeast, Mexico. Revista do Instituto de Medicina Tropical de São Paulo, 38(1), 29–33. 8762636 10.1590/s0036-46651996000100006

[105] Rego FD, Rugani JM, Shimabukuro PH, Tonelli GB, Quaresma PF, Gontijo CM. 2015. Molecular detection of *Leishmania* in phlebotomine sand flies (Diptera: Psychodidae) from a cutaneous leishmaniasis focus at Xakriaba Indigenous Reserve, Brazil. PLoS One, 10(4), e0122038. 25853254 10.1371/journal.pone.0122038 PMC4390197

[106] Rego FD, Shimabukuro PH, Quaresma PF, Coelho IR, Tonelli GB, Silva KM, Barata RA, Dias ES, Gontijo CM. 2014. Ecological aspects of the Phlebotominae fauna (Diptera: Psychodidae) in the Xakriaba Indigenous Reserve, Brazil. Parasites & Vectors, 7, 220. 24886717 10.1186/1756-3305-7-220 PMC4028289

[107] Rego FD, Souza GD, Miranda JB, Peixoto LV, Andrade-Filho JD. 2020. Potential vectors of *Leishmania* parasites in a recent focus of visceral leishmaniasis in neighborhoods of Porto Alegre, State of Rio Grande do Sul, Brazil. Journal of Medical Entomology, 57(4), 1286–1292. 32112089 10.1093/jme/tjaa036

[108] Resadore F, Júnior AMP, de Paulo PFM, Gil LHS, Rodrigues MMdS, Araújo MdS, Julião GR, Medeiros JF. 2018. Composition and vertical stratification of phlebotomine sand fly fauna and the molecular detection of *Leishmania* in forested areas in Rondônia State municipalities, Western Amazon, Brazil. Vector-Borne and Zoonotic Diseases, 19(5), 347–357. 30513068 10.1089/vbz.2018.2372

[109] Rigg CA, Calzada JE, Saldana A, Perea M, Chaves LF, Valderrama A. 2019. *Leishmania* spp. infection rate and feeding patterns of sand flies (Diptera: Psychodidae) from a hyperendemic cutaneous leishmaniasis community in Panama. American Journal of Tropical Medicine and Hygiene, 100(4), 798–807. 30793681 10.4269/ajtmh.17-0628 PMC6447106

[110] Rocha LS, Falqueto A, dos Santos CB, Ferreira AL, da Graca GC, Grimaldi G Jr., Cupolillo E. 2010. Survey of natural infection by *Leishmania* in sand fly species collected in southeastern Brazil. Transactions of the Royal Society of Tropical Medicine and Hygiene, 104(7), 461–466. 20346478 10.1016/j.trstmh.2010.02.005

[111] Rodriguez N, Aguilar CM, Barrios MA, Barker DC. 1999. Detection of *Leishmania braziliensis* in naturally infected individual sandflies by the polymerase chain reaction. Transactions of the Royal Society of Tropical Medicine and Hygiene, 93(1), 47–49. 10.1016/s0035-9203(99)90176-1 10492789

[112] Rosa J, Pereira DP, Brazil RP, Filho JD, Salomon O, Szelag E. 2012. Natural infection of *cortelezzii* complex (Diptera: Psychodidae: Phlebotominae) with *Leishmania braziliensis* in Chaco, Argentina. Acta Tropica, 123(2), 128–131. 22569560 10.1016/j.actatropica.2012.04.008

[113] Rowton E, de Mata M, Rizzo N, Navin T, Porter C. 1991. Vectors of *Leishmania braziliensi*s in the Peten, Guatemala. Parassitologia, 33(Suppl), 501–504. 1841251

[114] Rowton ED, de Mata M, Rizzo N, Porter CH, Navin TR. 1992. Isolation of *Leishmania braziliensis* from *Lutzomyia ovallesi* (Diptera:Psychodidae) in Guatemala. American Journal of Tropical Medicine and Hygiene, 46(4), 465–468. 1575293 10.4269/ajtmh.1992.46.465

[115] Ryan L, Fraiha H, Lainson R, Shaw JJ. 1986. New phlebotomine sandflies of the *walkeri* group (Diptera: Psychodidae) from Pará State, Brazil, with a pictorial key 1. Memórias do Instituto Oswaldo Cruz, 81(3), 323–331.

[116] Sanchez-Garcia L, Berzunza-Cruz M, Becker-Fauser I, Rebollar-Tellez EA. 2010. Sand flies naturally infected by *Leishmania* (*L*.) *mexican*a in the peri-urban area of Chetumal city, Quintana Roo, Mexico. Transactions of the Royal Society of Tropical Medicine and Hygiene, 104(6), 406–411. 20171709 10.1016/j.trstmh.2010.01.010

[117] Sandoval-Ramirez CM, Hernandez C, Teheran AA, Gutierrez-Marin R, Martinez-Vega RA, Morales D, Hoyos-Lopez R, Araque-Mogollon A, Ramirez JD. 2020. Complex ecological interactions across a focus of cutaneous leishmaniasis in Eastern Colombia: novel description of *Leishmania* species, hosts and phlebotomine fauna. Royal Society Open Science, 7(7), 200266. 32874625 10.1098/rsos.200266 PMC7428272

[118] Santamaria E, Ponce N, Zipa Y, Ferro C. 2006. Presence of infected vectors of *Leishmania* (*V*.) *panamensis* within dwellings in two endemic foci in the foothill of the middle Magdalena valley, western Boyaca, Colombia. Biomedica, 26(Suppl 1), 82–94. 17361845

[119] Santos TVD, Silveira FT. 2020. Increasing putative vector importance of *Trichophoromyi*a phlebotomines (Diptera: Psychodidae). Memórias do Instituto Oswaldo Cruz, 115, e190284. 32049097 10.1590/0074-02760190284 PMC7012582

[120] Saraiva L, Andrade Filho JD, Silva Sde O, Andrade AS, Melo MN. 2010. The molecular detection of different *Leishmania* species within sand flies from a cutaneous and visceral leishmaniasis sympatric area in Southeastern Brazil. Memórias do Instituto Oswaldo Cruz, 105(8), 1033–1039. 21225201 10.1590/s0074-02762010000800013

[121] Saraiva L, Carvalho DA, Gontijo GM, Quaresma PF, Lima AC, Falcao AL, Andrade Filho JD. 2009. Natural infections *Lutzomyia neivai* and *Lutzomyia sallesi* (Dipera: Psychodidae) by *Leishmania infantum chagasi* in Brazil. Journal of Medical Entomology, 46(5), 1159–1163. 19769049 10.1603/033.046.0525

[122] Savani ES, Nunes VL, Galati EA, Castilho TM, Zampieri RA, Floeter-Winter LM. 2009. The finding of *Lutzomyia almerioi* and *Lutzomyia longipalpis* naturally infected by *Leishmani*a spp. in a cutaneous and canine visceral leishmaniases focus in Serra da Bodoquena, Brazil. Veterinary Parasitology, 160(1–2), 18–24. 19062193 10.1016/j.vetpar.2008.10.090

[123] Shaw JJ, Ishikawa EA, Lainson R, Braga RR, Silveira FT. 1991. Cutaneous leishmaniasis of man due to *Leishmania* (*Viannia*) *shaw*i Lainson, de Souza, Povoa, Ishikawa & Silveira, in Para State, Brazil. Annales de Parasitologie Humaine et Comparée, 66(6), 243–246. 1822654 10.1051/parasite/1991666243

[124] Shaw JJ, Lainson R. 1968. Leishmaniasis in Brazil: II. Observations on enzootic rodent leishmaniasis in the Lower Amazon Region – the feeding habits of the vector, *Lutzomyia flaviscutellata* in reference to man, rodents and other animals. Transactions of the Royal Society of Tropical Medicine and Hygiene, 62(3), 396–405. 5659233 10.1016/0035-9203(68)90091-6

[125] Shaw JJ, Lainson R, Ward RD. 1972. Leishmaniasis in Brazil. VII. Further observations on the feeding habitats of *Lutzomyia flaviscutellata* (Mangabeira) with particular reference to its biting habits at different heights. Transactions of the Royal Society of Tropical Medicine and Hygiene, 66(5), 718–723. 4647643 10.1016/0035-9203(72)90085-5

[126] Silva ANR, Júnior AMP, de Paulo PFM, da Silva MS, Castro TS, Costa GdS, Freitas MTdS, Rodrigues MMdS, Medeiros JF. 2021. Detection of *Leishmania* species (Kinetoplastida, Trypanosomatidae) in phlebotomine sand flies (Diptera, Psychodidae) from Porto Velho, Northern Brazil. Acta Tropica, 213, 105757. 33189711 10.1016/j.actatropica.2020.105757

[127] Silva TR, Assis MD, Freire MP, Rego FD, Gontijo CM, Shimabukuro PH. 2014. Molecular detection of *Leishmania* in sand flies (Diptera: Psychodidae: Phlebotominae) collected in the caititu indigenous reserve of the municipality of Labrea, State of Amazonas, Brazil. Journal of Medical Entomology, 51(6), 1276–1282. 26309318 10.1603/ME14025

[128] Silveira FT, Shaw JJ, Braga RR, Ishikawa E. 1987. Dermal leishmaniasis in the Amazon region of Brazil: *Leishmania* (*Viannaia*) *lainsoni* sp.n., a new parasite from the State of Pará. Memórias do Instituto Oswaldo Cruz, 82(2), 289–291. 3506634 10.1590/s0074-02761987000200018

[129] Silveira FT, Souza AA, Lainson R, Shaw JJ, Braga RR, Ishikawa EE. 1991. Cutaneous leishmaniasis in the Amazon region: natural infection of the sandfly *Lutzomyia ubiquitalis* (Psychodidae: Phlebotominae) by *Leishmania* (*Viannia*) *lainson*i in Para State, Brazil. Memórias do Instituto Oswaldo Cruz, 86(1), 127–130. 10.1590/s0074-02761991000100021 1842393

[130] Souza NA, Andrade-Coelho CA, Vilela ML, Peixoto AA, Rangel EF. 2002. Seasonality of *Lutzomyia intermedia* and *Lutzomyia whitmani* (Diptera: Psychodidae: Phlebotominae), occurring sympatrically in area of cutaneous leishmaniasis in the State of Rio de Janeiro, Brazil. Memórias do Instituto Oswaldo Cruz, 97(6), 759–765. 12386692 10.1590/s0074-02762002000600001

[131] SUCEN. 2005. Encontro de *Lutzomyia edwardsi* infectada na região da Grande São Paulo. Revista de Saúde Pública, 39(1), 137–138. 15654471 10.1590/s0034-89102005000100018

[132] Tanure A, Rêgo FD, Tonelli GB, Campos AM, Shimabukuro PHF, Gontijo CMF, Paz GF, Andrade-Filho JD. 2020. Diversity of phlebotomine sand flies and molecular detection of trypanosomatids in Brumadinho, Minas Gerais, Brazil. PLoS One, 15(6), e0234445. 32579586 10.1371/journal.pone.0234445 PMC7314019

[133] Teles CB, Santos AP, Freitas RA, Oliveira AF, Ogawa GM, Rodrigues MS, Pessoa FA, Medeiros JF, Camargo LM. 2016. Phlebotomine sandfly (Diptera: Psychodidae) diversity and their *Leishmania* DNA in a hot spot of american cutaneous leishmaniasis human cases along the Brazilian border with Peru and Bolivia. Memórias do Instituto Oswaldo Cruz, 111, 423–432. 10.1590/0074-02760160054 PMC4957494 27304023

[134] Teodoro U, La Salvia Filho V, de Lima EM, Spinosa RP, Barbosa OC, Ferreira ME, Lonardoni MV. 1993. Observações sobre o comportamento de flebotomíneos em ecotopos florestais e extraflorestais, em area endemica de leishmaniose tegumentar americana, no norte de Estado do Parana, sul do Brasil. Revista de Saúde Pública, 27(4), 242–249. 8209155 10.1590/s0034-89101993000400003

[135] Tesh RB, Chaniotis BN, Aronson MD, Johnson KM. 1971. Natural host preferences of Panamanian sandflies as determined by precipitin test. American Journal of Tropical Medicine and Hygiene, 20, 150–156. 5567741 10.4269/ajtmh.1971.20.150

[136] Torrez M, Lopez M, Le Pont F, Martinez E, Munoz M, Hervas D, Yaksic N, Arevalo J, Sossa D, Dedet JP, Dujardin JP. 1998. *Lutzomyia nuneztovari anglesi* (Diptera: Psychodidae) as a probable vector of *Leishmania braziliensis* in the Yungas, Bolivia. Acta Tropica, 71(3), 311–316. 9879740 10.1016/s0001-706x(98)00068-0

[137] Travi BL, Vélez ID, Brutus L, Segura I, Jaramillo C, Montoya J. 1990. *Lutzomyia evansi*, an alternate vector of *Leishmania chagasi* in a Colombian focus of visceral leishmaniasis. Transactions of the Royal Society of Tropical Medicine and Hygiene, 84(5), 676–677. 2278068 10.1016/0035-9203(90)90142-2

[138] Uzcátegui Y, Vasconcelos dos Santos T, Ramos P, Santos E, Póvoa M. 2020. Phlebotomines (Diptera: Psychodidae) from a Urban Park of Belém, Pará State, Northern Brazil and Potential Implications in the Transmission of American Cutaneous Leishmaniasis. Journal of Medical Entomology, 57(1), 281–288. 31550368 10.1093/jme/tjz153 PMC6951032

[139] Valdivia HO, De Los Santos MB, Fernandez R, Baldeviano GC, Zorrilla VO, Vera H, Lucas CM, Edgel KA, Lescano AG, Mundal KD, Graf PC. 2012. Natural *Leishmania* infection of *Lutzomyia auraensis* in Madre de Dios, Peru, detected by a fluorescence resonance energy transfer-based real-time polymerase chain reaction. American Journal of Tropical Medicine and Hygiene, 87(3), 511–517. 22802444 10.4269/ajtmh.2012.11-0708 PMC3435357

[140] Vasconcelos Dos Santos T, de Pita-Pereira D, Araujo-Pereira T, Britto C, Silveira FT, Povoa MM, Rangel EF. 2019. *Leishmania* DNA detection and species characterization within phlebotomines (Diptera: Psychodidae) from a peridomicile-forest gradient in an Amazonian/Guianan bordering area. PLoS One, 14(7), e0219626. 31306447 10.1371/journal.pone.0219626 PMC6629084

[141] Vasquez Trujillo A, Gonzalez Reina AE, Gongora Orjuela A, Prieto Suarez E, Palomares JE, Buitrago Alvarez LS. 2013. Seasonal variation and natural infection of *Lutzomyia antunesi* (Diptera: Psychodidae: Phlebotominae), an endemic species in the Orinoquia region of Colombia. Memórias do Instituto Oswaldo Cruz, 108(4), 463–469. 23828011 10.1590/0074-0276108042013011 PMC3970617

[142] Vasquez-Trujillo A, Santamaria-Herreno E, Gonzalez-Reina AE, Buitrago-Alvarez LS, Gongora-Orjuela A, Cabrera-Quintero OL. 2008. *Lutzomyia antunesi* as suspected vector of cutaneous leishmaniasis in the Orinoquian region of Colombia. Revista de Salud Pública (Bogota), 10(4), 625–632. 10.1590/s0124-00642008000400012 19360212

[143] Vigoder FM, Souza NA, Brazil RP, Bruno RV, Costa PL, Ritchie MG, Klaczko LB, Peixoto AA. 2015. Phenotypic differentiation in love song traits among sibling species of the *Lutzomyia longipalpi*s complex in Brazil. Parasites & Vectors, 8(1), 290. 26017472 10.1186/s13071-015-0900-8 PMC4456791

[144] Villaseca P, Llanos-Cuentas A, Perez E, Davies CR. 1993. A comparative field study of the relative importance of *Lutzomyia peruensis* and *Lutzomyia verrucarum* as vectors of cutaneous leishmaniasis in the Peruvian Andes. American Journal of Tropical Medicine and Hygiene, 49(2), 260–269. 8357089 10.4269/ajtmh.1993.49.260

[145] Warburg A, Montoya-Lerma A, Jaramillo C, Cruz-Ruiz AL, Ostrovska K. 1991. Leishmaniasis vector potential of *Lutzomyia* spp. in Colombian coffee plantations. Medical and Veterinary Entomology, 5(1), 9–16. 1768906 10.1111/j.1365-2915.1991.tb00514.x

[146] Ward RD, Phillips A, Burnet A, Marcondes CR. 1988. The *Lutzomyia longipalpis* complex: reproduction and distribution, in Biosystematics of haematophagus insects, Service MW, Editor. Clarendon Press: Oxford, p. 257–269.

[147] Ward RD, Shaw JJ, Lainson R, Fraiha H. 1973. Leishmaniasis in Brazil: VIII. Observations on the phlebotomine fauna of an area of highly endemic cutaneous leishmaniasis in the Serra dos Carajás, Pará State. Transactions of the Royal Society of Tropical Medicine and Hygiene, 67, 174–183. 4784054 10.1016/0035-9203(73)90142-9

[148] Young DG, Duncan MA. 1994. Guide to the identification and geographic distribution of *Lutzomyia* sand flies in Mexico, the West Indies, Central and South America (Diptera: Psychodidae). Memoirs of the American Entomological Institute, 54, 1–881.

[149] Young DG, Morales A, Kreutzer RD, Alexander JB, Corredor A, Tesh RB, Ferro de Carrasquilla C, de Rodriguez C. 1987. Isolations of *Leishmania braziliensis* (Kinetoplastida: Trypanosomatidae) from cryopreserved Colombian sand flies (Diptera: Psychodidae). Journal of Medical Entomology, 24(5), 587–589. 3669032 10.1093/jmedent/24.5.587

[150] Zorrilla V, De Los Santos MB, Espada L, Santos RDP, Fernandez R, Urquia A, Stoops CA, Ballard SB, Lescano AG, Vasquez GM, Valdivia HO. 2017. Distribution and identification of sand flies naturally infected with *Leishmania* from the Southeastern Peruvian Amazon. PLoS Neglected Tropical Diseases, 11(11), e0006029. 29107954 10.1371/journal.pntd.0006029 PMC5673161

